# Machine learning reveals hidden stability code in protein native fluorescence

**DOI:** 10.1016/j.csbj.2021.04.047

**Published:** 2021-04-28

**Authors:** Hongyu Zhang, Yang Yang, Cheng Zhang, Suzanne S. Farid, Paul A. Dalby

**Affiliations:** a Department of Biochemical Engineering, UCL, London WC1E 6BT, UK; b EPSRC Future Targeted Healthcare Manufacturing Hub, UCL, London WC1E 6BT, UK

**Keywords:** Protein stability, Machine learning, Biopharmaceuticals

## Abstract

Conformational stability of a protein is usually obtained by spectroscopically measuring the unfolding melting temperature. However, optical spectra under native conditions are considered to contain too little resolution to probe protein stability. Here, we have built and trained a neural network model to take the temperature-dependence of intrinsic fluorescence emission under native-only conditions as inputs, and then predict the spectra at the unfolding transition and denatured state. Application to a therapeutic antibody fragment demonstrates that thermal transitions obtained from the predicted spectra correlate highly with those measured experimentally. Crucially, this work reveals that the temperature-dependence of native fluorescence spectra contains a high-degree of previously hidden information relating native ensemble features to stability. This could lead to rapid screening of therapeutic protein variants and formulations based on spectroscopic measurements under non-denaturing temperatures only.

## Graphical abstract

**Figure:**
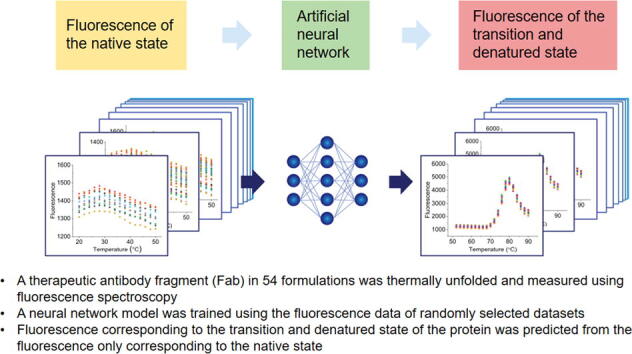

## 1 Introduction

The measurement of conformational stability is crucial in protein folding study, as well as for the engineering and formulation development of protein-based therapeutics [1], [2], [3]. Proteins are marginally stable with their tertiary structures formed by many weak non-covalent interactions, such that a small change in buffer composition or temperature can lead to unfolding and aggregation. The thermodynamic stability of proteins is often determined by monitoring the fraction of protein unfolding as a function of step-wise increases in temperature or chemical denaturant, giving rise to a transition phase that defines a thermal- or chemical-denaturation mid-point (*T*_m_ or *C*_m_) [4]. These measures are often used as rapid screens for improving the conformational stability of proteins through mutagenesis or formulation of the buffer conditions, aiming at a protein variant or formulation with improved kinetic stability to unfolding or aggregation at a given storage temperature. In some cases, a buffer increasing the *T*_m_ also makes the protein more kinetically stable [5]. Recently, mutations of an antibody fragment led to improved aggregation kinetics in cases that decreased the native ensemble flexibility, yet without altering the *T*_m_ [6].

The known influence of local unfolding, conformational flexibility and protein–protein interactions within the native structure ensemble, on the conformational stability of proteins, as measured by their *T*_m_ and their propensity to aggregate, led us to examine whether spectra of proteins under native conditions (at temperatures much lower than the *T*_m_) contain sufficient information to predict their thermal unfolding transitions at higher temperatures. Indeed, it has been observed previously that the intrinsic fluorescence in the baseline of denaturation curves at lower temperatures or lower denaturant concentrations, for thermal and chemical denaturation experiments respectively, is sensitive to changes in the buffer [7]. For example, with increasing guanidinium concentration, the slope of the baseline became steeper which was postulated that the local structure of the tryptophan residues was rearranged prior to the major unfolding of the protein.

Machine learning (ML) refers to a series of algorithms capable of identifying underlying patterns, features and relationships between various variables from complex datasets such that robust prediction models can be built [8], [9], [10]. In biological studies, ML has shown great potential to analyze the genomics [11], [12] and proteomics [13], [14], [15] data. Recently, ML has been applied also to predict the infrared spectra of proteins [16] and the impact of sequence mutations or buffer compositions on protein stability and aggregation kinetics [17], [18], [19], [20], [21]. However, none of these studies has attempted to use the spectral data of the native protein generated under different buffer conditions as the input variables from which to predict its conformational stability.

**Fig. 1:**
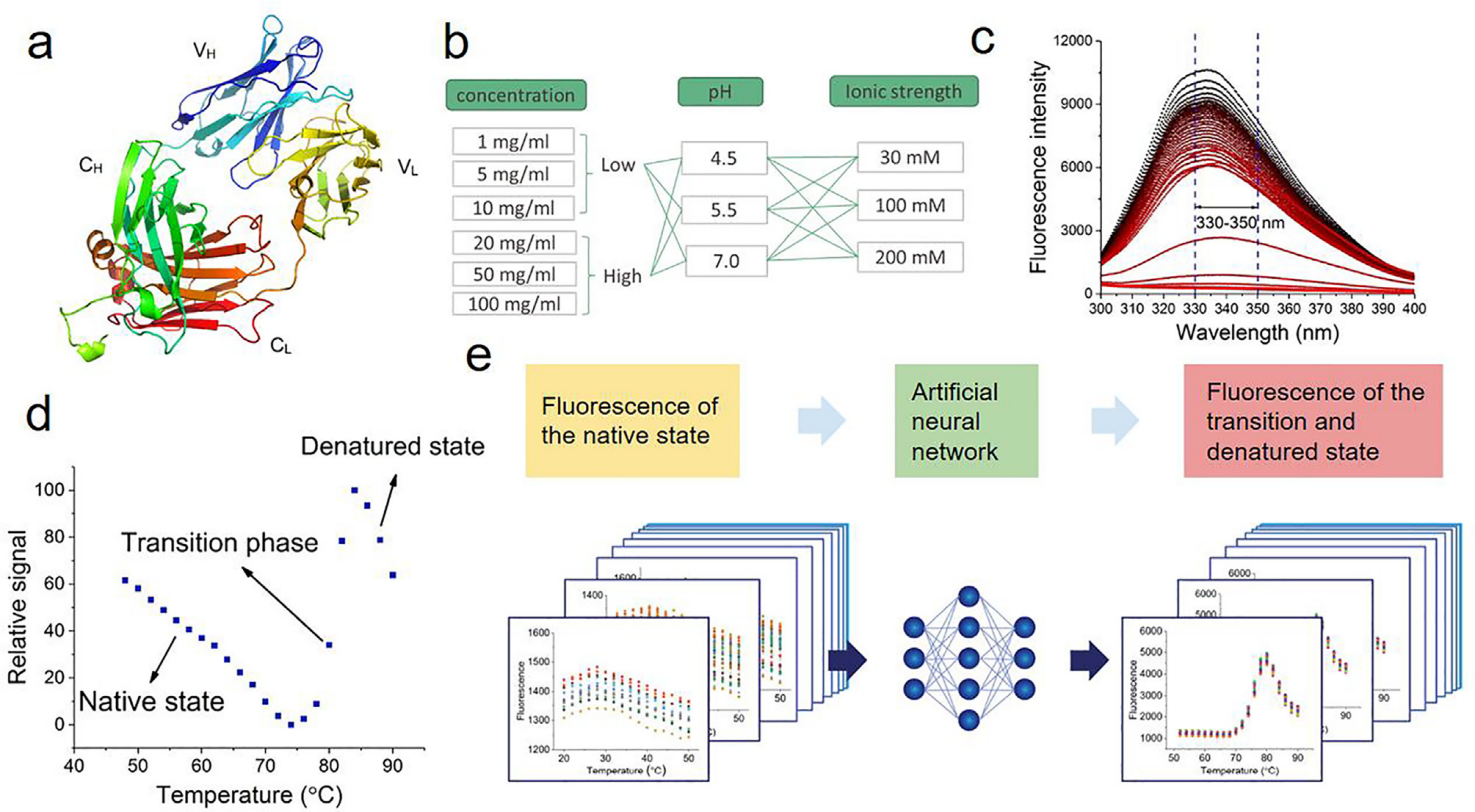
(a) The original denaturation fluorescence data (wavelength 330-350 nm) measured for 54 experiment conditions. (b) The performance of Feedforward Neural Network model with one hidden layerof 20 neurons using Matlab (R2017a). After 208 epochs the training stopped when the validation check was met.

Here, we perform an experimental and ML analysis of the temperature-dependent intrinsic fluorescence spectra of a therapeutic antibody fragment for antigen-binding (Fab) over industrially-relevant experimental conditions including six protein concentrations at 1–100 mg/mL, three pH values from 4.5 to 7.0, and three ionic strengths (IS) spanning 30–200 mM (Fig. 1). We obtained the full thermal denaturation curves for each of the 54 experimental conditions, whereby the fluorescence emission intensity was measured over a wide range of wavelengths (300–400 nm), at 0.5 nm intervals. Using a subset of the fluorescence spectra (330–350 nm), which has the maximal changes upon unfolding, a neural network algorithm was applied to model the curves in the transition and denatured regions from only the spectral data in the native baseline regions at lower temperatures. The entire modelled curves were then each fitted to a two-state unfolding model for comparison to equivalent fits obtained directly with the experimental data. In doing so, we interrogated whether the baseline spectral data, which correspond to the native state of the protein structure, contain sufficient information to enable a robust prediction of the denaturation profile and associated conformational stability of the protein. This was based on the hypothesis that the native structure ensemble and hence spectral data represent fingerprints of underlying characteristics such as local unfolding, conformational flexibility and protein–protein interactions. The ML-modelled denaturation curves and the derived *T*_m_ showed a good consistency with the experimental results. This suggests that it is possible to reveal hidden information within the native-state fluorescence spectra at lower temperatures to predict the transition and denaturation profiles for unfolding.

## 2 Materials and methods

### 2.1 Materials

The *E. coli* strain W3110 containing pTTOD plasmid for Fab expression was obtained from UCB (Slough, UK). A C226S variant of Fab was used as described previously, to eliminate any dimerization through disulphide bond formation [22]. All reagents including buffers and inorganic salts are analytical grade and purchased from Sigma-Aldrich (Poole, UK).

### 2.2 Protein preparation

Fab was produced in a pilot-scale 30 L fermenter (BIOSTAT Cplus, Sartorius, Goettingen, Germany) and purified using AKTA-based liquid chromatography as described elsewhere [22]. The purified protein was then exchanged to respective buffers: sodium acetate was used to prepare pH 4.5 and pH 5.5 buffers, and sodium phosphate was used to prepare pH 7.0 buffer, to make the initial ionic strength of the buffers were 30 mM. The protein was then concentrated to make 1, 5, 10, 20, 50, 100 mg/mL solutions with the final ionic strength adjusted to 100 mM or 200 mM using 1 M sodium chloride.

### 2.3 Thermal denaturation measurement

The thermal stability of Fab was measured using a UNit system (UNCHAINED LABS, Pleasanton, US). 9 μL of the protein was loaded into the sample well in a 16-well cartridge. The cartridge was loaded into the instrument and equilibrated to 20 °C prior to being step-heated from 20 to 90 °C at 30 s per 2 °C interval. The intrinsic fluorescence spectrum at each temperature was recorded for 3 independent samples from 250 to 725 nm. The static light scattering (SLS) of the sample was concurrently collected by the instrument for 266 and 473 nm, which corresponds to the formation of small and large aggregates, respectively.

### 2.4 Fitting of the denaturation curve

The fluorescence intensity at 340 nm for each experiment or model-predicted curve was extracted and plotted against temperature, then fitted to a two-state unfolding model to obtain the midpoint of unfolding transitions (*T*_m_), as described previously [6]. In most cases, the fluorescence data below 330 K (57 °C) and over 363 K (90 °C) were removed to improve the fits to the transition region, by using the most linear portions of the baselines:

(1) $$I_{T}=\frac{\left(I_{N}+aT\right)+\left(I_{D}+bT\right)K}{1+K}$$

where *K* is the equilibrium constant for the transition between the native and denatured state; *T* is the experimental temperature; *I_T_*, *I_N_* and *I_D_* are the spectroscopic signals of the protein at each given temperature, at the native and at the fully denatured state, respectively. *a* and *b* are the baseline slopes of the native and denatured region of the curve.

[2] $$K=\exp\left[\frac{\Delta H_{\mathrm{vh}}}{R}\left(\frac{1}{T_{m}}-\frac{1}{T}\right)\right]$$

where *T_m_* is the temperature at which the protein is half denatured; *ΔH_vn_* is the van’t Hoff enthalpy and *R* is the gas constant. All temperature terms in this equation are absolute temperatures in *Kelvin*.

To obtain *T_m_* individually from each 1 nm between 330 and 350 nm, the denaturation curves of each wavelength were globally fitted to the two-state model by sharing the *ΔH_vh_* but varying *T_m_* values. The obtained *T_m_* values were plotted against native slope baseline or initial fluorescence at 20 °C (Figure S6 & S7).

### 2.5 Machine learning of the thermal denaturation data

Artificial neural network (ANN) algorithms are a type of machine learning (ML), inspired by human neural networks, which result in data-driven models that can interpret effectively patterns in multivariate data from non-linear systems [23]. In this study, a common ANN algorithm, Feedforward Neural Network (FFNN) was applied to construct models with one hidden layer of 20 neurons using Matlab (R2017a).

For each epoch, the training set was used to train the neural network model by fitting the weights of connections between neurons while the current model was evaluated by the test set and adjusted according to the test result. The validation dataset provided an unbiased evaluation of the model fit on the training dataset. When the whole training procedure was completed, the model with the best performance from the validation set was selected as the final optimal neural network model.

The maximum number of epochs to train was set to 1000. The performance of the trained network was assessed by the mean squared error (MSE) function and the performance goal as expected MSE of the model was set as 20,000 (based on 1.5% error of the average fluorescence data). To prevent the trained network model from over-training, the training procedure stops if the validation performance degrades for 10 consecutive epochs and the optimal trained network with the best validation performance is selected. The training function used in this work to construct FFNN was the Levenberg-Marquardt algorithm, which is designed to solve non-linear least squares problems [24]. The Levenberg-Marquardt algorithm uses the Jacobian matrix in the following Newton-like model:

(3) $$x_{k+1}=x_{k}-{\left[J^{T}J+\mu I\right]}^{-1}J^{T}e$$

where *J* is the Jacobian matrix that contains first derivatives of the network errors with respect to the weights and biases, and *e* is a vector of network errors. If the scalar *µ* is zero, this is just Newton's method using the approximate Hessian matrix. If *µ* is large, this becomes gradient descent with a small step size. Thus, *µ* is decreased after each successful step and is increased only when a tentative step would increase the performance function. The activation functions for the hidden layer and output layer are the hyperbolic tangent sigmoid transfer function and linear transfer function, respectively.

A total of 2268 thermal denaturation measurement data, including protein concentration, pH, IS, wavelength and native state fluorescence intensity at each 0.5 nm of 330–350 nm, were used as inputs to feed the training model. The output of the model was the high temperature (52–90 °C) section of the denaturation curve corresponding to the transition and denatured state of the protein.

Cross-validation of the ML models was achieved with one of the simplest and commonly-used techniques known as the holdout method [25]. This method was suitable for this work since it permitted the same training, test and validation sets to be used for building different models and therefore made it easier to compare the predicted *T*_m_ values across models derived from different input ranges. To avoid the potential risks of overfitting and selection bias with the holdout method, the splitting strategy was designed to split the total thermal denaturation measurement data into training, validation and test sets in the ratio of 66%, 17% and 17% and to guarantee an even spatial distribution throughout the experimental conditions. More specifically, the six protein concentrations were split into two categories, low concentration (1, 5 and 10 mg/ml) and high concentration (20, 50 and 100 mg/ml) (Fig. 1b). Given the two concentration categories, three pH values and three ionic strength values, a total of 18 combinations resulted. Then, from each combination, the method picked two concentration values from a set of three values as the training set, while the other concentration was allocated to either the validation set or test set. In this case, there were 3 runs of cross-validation to make sure all experimental conditions were used for both training and test/validation and that each condition was used for test/validation exactly once. The performances (measured in MSE) for the three runs of cross-validation were 22043, 23778 and 25309 and the splitting strategy with the best performance (as shown in Table 1) was selected for model building in this work.

**Table 1:** Splitting strategy of data set into training, validation and test dataset for Neural Network model.

Concentration (mg/mL)	pH	IS (mM)	Division	Concentration (mg/mL)	pH	IS (mM)	Division
1	4.5	30	Validation	20	4.5	30	Test
5			Training	50			Training
10			Training	100			Training
1	4.5	100	Training	20	4.5	100	Training
5			Test	50			Validation
10			Training	100			Training
1	4.5	200	Training	20	4.5	200	Training
5			Training	50			Training
10			Validation	100			Test
1	5.5	30	Test	20	5.5	30	Validation
5			Training	50			Training
10			Training	100			Training
1	5.5	100	Training	20	5.5	100	Training
5			Validation	50			Test
10			Training	100			Training
1	5.5	200	Training	20	5.5	200	Training
5			Training	50			Training
10			Test	100			Validation
1	7	30	Validation	20	7	30	Test
5			Training	50			Training
10			Training	100			Training
1	7	100	Training	20	7	100	Training
5			Test	50			Validation
10			Training	100			Training
1	7	200	Training	20	7	200	Training
5			Training	50			Training
10			Validation	100			Test

In order to demonstrate that there was no overfitting bias with the holdout method, the FFNN modelling procedure was repeated using the k-fold cross validation method and their performance compared [25]. The thermal denaturation measurement data was randomly partitioned into k = 6 equal size subsets. Of the six subsets, two subsets were retained as the validation set and test set respectively, and the remaining four subsets used as training data. The cross-validation process was then repeated six times (the folds), with each of the six subsamples used exactly once as the validation data and test data. This work chose k = 6 to make sure the ratio of training, validation and test sets were 66%, 17% and 17% so as to match the ratio in the holdout method. The performance (measured as MSE) of the k-fold cross-validation had an average MSE of 24,333 compared to 22,043 for the holdout method. The similar performance demonstrated that the splitting strategy applied in the holdout method avoided overfitting or bias issues. The holdout method had the additional benefit of saving time to retain a similar performance result.

## 3 Results

### 3.1 Thermal denaturation measurement of Fab

The denaturation of Fab results in the exposure of the tryptophan residues that lead to a change in fluorescence intensity as a function of temperature. The fluorescence at 340 nm was selected and fitted to a two-state model for each protein concentration and buffer condition (Fig. 3 a1-6). The temperature at which the protein was half-denatured (*T*_m_) was obtained from the fit and shown in Table S1. Previously, 1 mg/ml Fab was observed to aggregate rapidly at above the *T*_m_, whereby the thermal unfolding transition was a convolution of the conformational unfolding equilibrium, and the aggregation kinetics [22]. Thus, the precise *T*_m_ value obtained is affected by the experimental settings, particularly the ramp rate of the thermal denaturation, which is accordingly kept constant across all experiments.

The change in *T*_m_ with increasing protein concentration shows stability variation across the experimental conditions, showing a convergence towards a low variability at 100 mg/mL (Figure S1). This could be partially owing to the quality of data for the fit as the fitting error obtained for *T*_m_ at 1–10 mg/mL is generally larger than those of 20–100 mg/mL data, which have better unfolding transitions to determine the *T*_m_. However, if a comparison is made within the 20–100 mg/mL group, a smaller variation in *T*_m_ over different buffers is still observed for 100 mg/mL suggesting that the *T*_m_ of Fab becomes relatively insensitive to changes in pH or ionic strength at 100 mg/mL.

The *T*_m_ has comparatively larger errors for Fab at 1–10 mg/mL than at higher concentrations. The light scattering of Fab shows the aggregates formed from the denatured state at 1 mg/mL rapidly precipitate out of the solution resulting in a drop of scattering intensity, whereas the size of aggregate formed at higher concentrations is still within the measurable range (Figure S5 & S6). This is consistent with previous results showing that the aggregation of Fab is kinetically (unpublished results) and also thermodynamically stabilized at the higher protein concentrations [26]. Therefore, at lower concentrations of Fab, the greater convolution of aggregation kinetics with the unfolding transition leads to the higher fitting error for *T*_m_.

### 3.2 Modeling of the denaturation curves from the native state fluorescence

A common artificial neural network algorithm, Feedforward Neural Network (FFNN), was applied and carried out with a splitting of the 2268 experimental datasets into 1512 (66%) for training, 378 (17%) for validation, 378 (17%) for testing (Table 1). The validation and test datasets were selected to obtain an even spatial distribution throughout the concentration, pH and IS conditions, so as to avoid overfitting or bias in the results (as described in Materials and Methods).The results of the k-fold cross-validation is shown in Figure S2. For each dataset, the fluorescence intensity between wavelength 330–350 nm at 0.5 nm intervals of the emission spectra were selected from the temperatures corresponding to the native state baseline of the protein. In the first model, each wavelength had 16 fluorescence intensity data points, corresponding to the 2 °C intervals spanning 20–50 °C (293–323 K). The fluorescence intensity data were combined with the protein concentration, pH, IS and wavelength information to form the 20 inputs defined as input range 1 (Table S2).

**Fig. 2:**
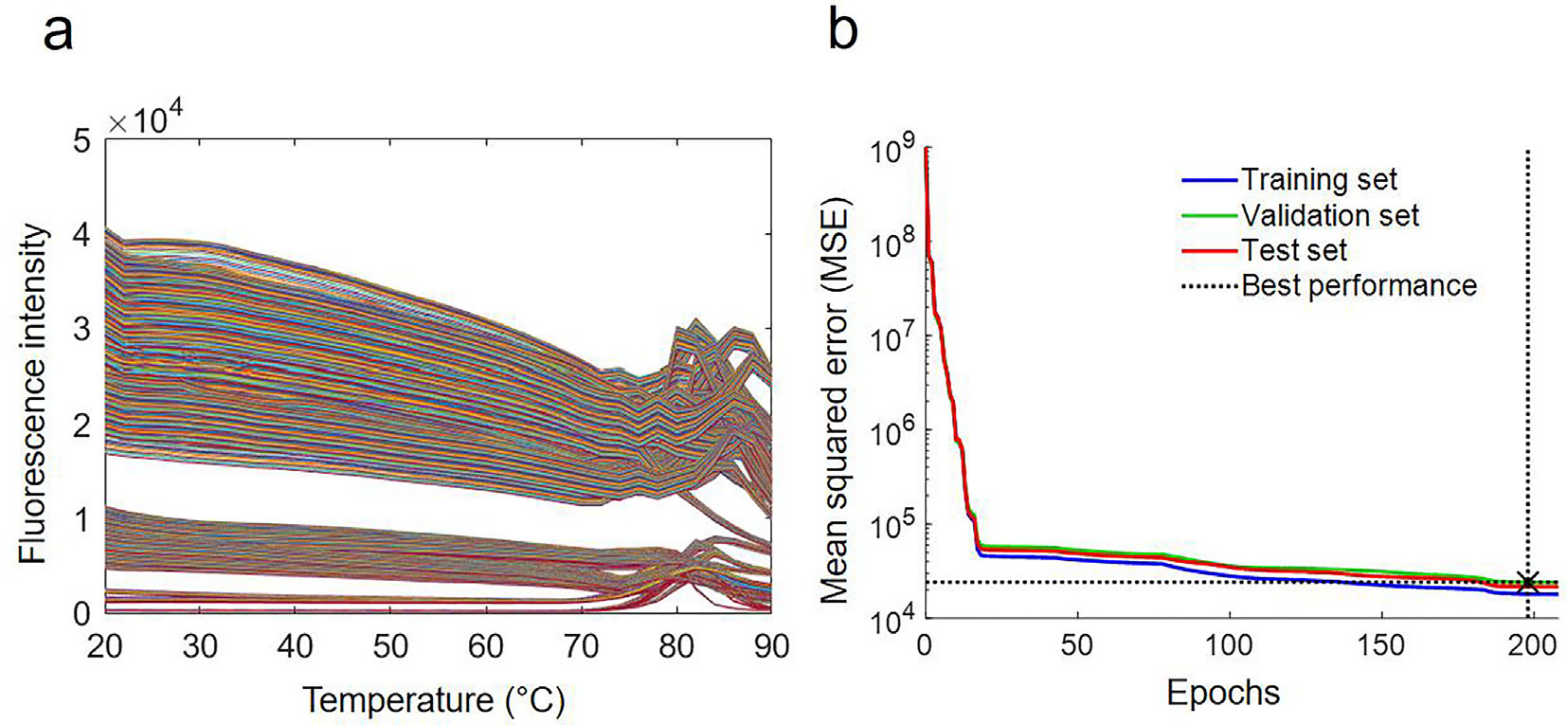
(a) The original denaturation fluorescence data (wavelength 330–350 nm) measured for 54 experiment conditions. (b) The performance of Feedforward Neural Network model with one hidden layer of 20 neurons using Matlab (R2017a). After 208 epochs the training stopped when the validation check was met.

The outputs of the model were the fluorescence intensities between 330 and 350 nm at 0.5 nm intervals for 52–90 °C spanning the transition region and denatured-state baseline (Figure S3). The neural network training performance was analyzed in terms of the mean squared error (MSE) of each epoch for the training, test and validation datasets, as shown in Fig. 2b. The MSE decreased with the increase in the number of epochs for all training, validation and test datasets. The optimal model was found at epoch194 with the best validation performance in terms of the lowest MSE value.

**Fig. 3:**
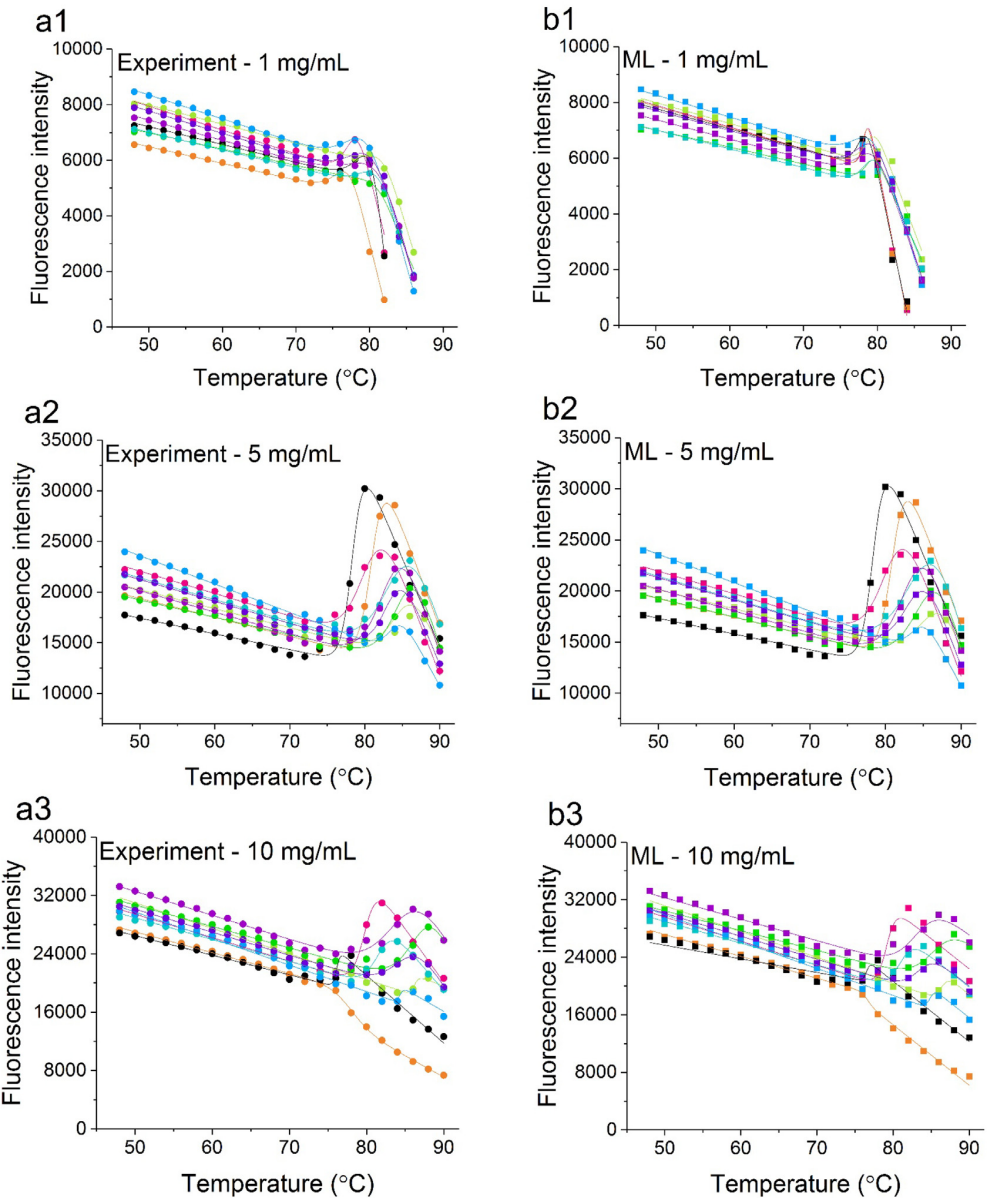

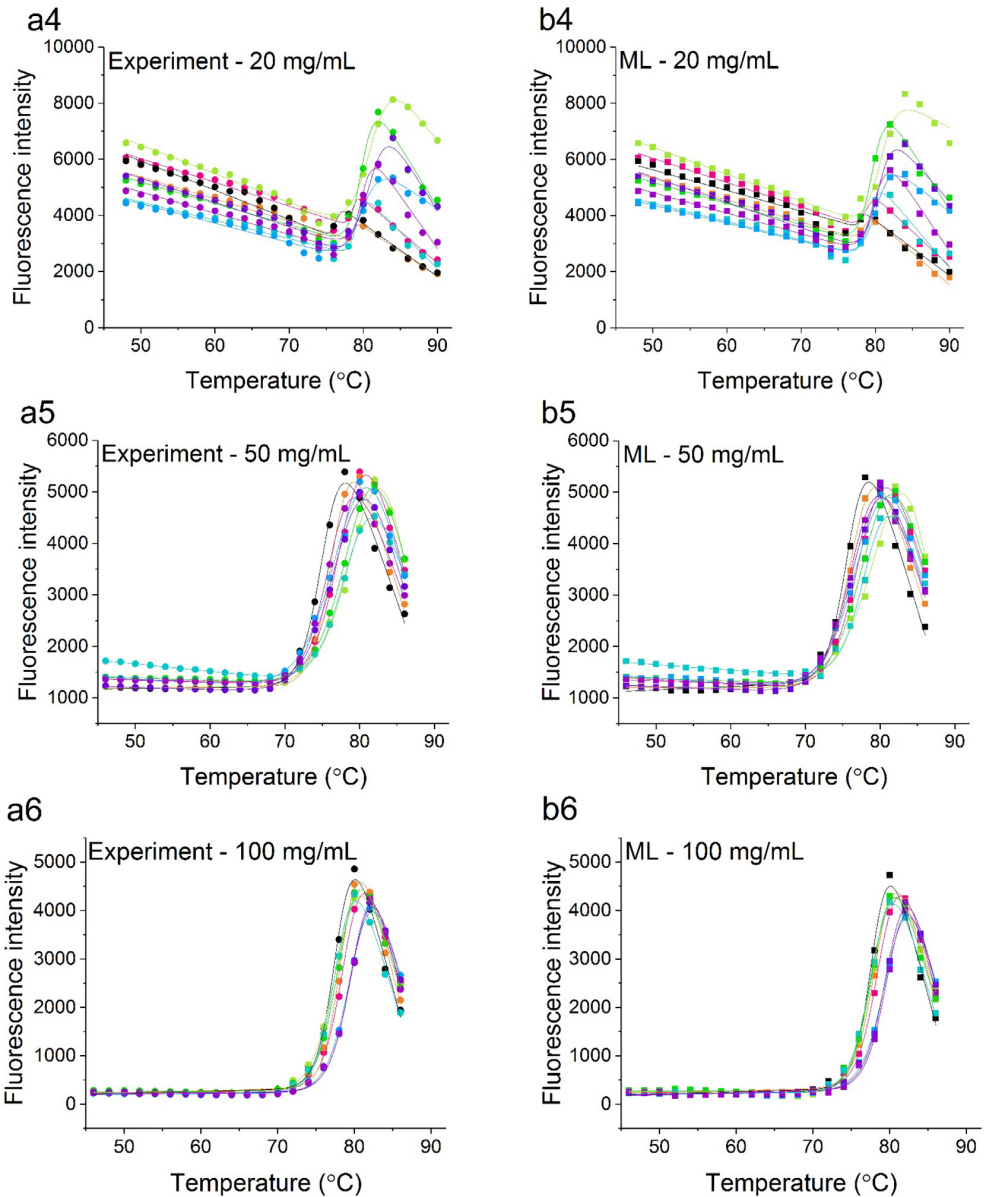
Thermal denaturation curves of Fab obtained by fluorescence measurements for (a1-6) 1 mg/mL, 5 mg/mL, 10 mg/mL, 20 mg/mL, 50 mg/mL and 100 mg/mL over 9 buffer conditions. The ML-derived fluorescence (training from 20 to 50 °C shown as example) over the same temperature range are shown in b1-6. The change in fluorescence intensity at 340 nm was plotted as filled circles (pH4.5 IS 30 mM: , pH4.5 IS 100 mM:  pH4.5 IS 200 mM: , pH5.5 IS 30 mM: , pH5.5 IS 100 mM: , pH5.5 IS 200 mM: , pH7.0 IS 30 mM: , pH7.0 IS 100 mM: , pH7.0 IS 200 mM: ) and squares (pH4.5 IS 30 mM: , pH4.5 IS 100 mM: , pH4.5 IS 200 mM: , pH5.5 IS 30 mM: , pH5.5 IS 100 mM: , pH5.5 IS 200 mM: , pH7.0 IS 30 mM: , pH7.0 IS 100 mM: , pH7.0 IS 200 mM: ) against temperature. The solid lines represent the best fit of the data to a two-state unfolding model to derive the *T*_m_.

The predicted output fluorescence data (52–90 °C) were plotted together with the experimental native-state baseline data used as the input (20–50 °C), to generate the complete denaturation curves (20–90 °C). Their native and denatured baselines were truncated in the same way as for the experimental data, to include only the linear regions of the baselines prior to fitting to the same two-state model (Fig. 3, Table S1).

In three subsequent models, to characterize the impact of input data volume on the quality of the output, we reduced the input fluorescence intensity range from 20 to 50 °C to 20–40 °C, and eventually to 20–30 °C. The predicted curves were also plotted as above, and then fitted to the two-state model (Figure S4). Moreover, to investigate whether the feature information in the native state baseline was evenly distributed in temperature or biased to a certain temperature range, we carried out the same modelling procedure but limited the input fluorescence intensity for smaller scanning windows of 20–30 °C (as above), 30–40 °C or 40–50 °C (Figure S3). Finally, to investigate the reliance of the models upon experimental condition information (concentration, pH and IS), we removed these parameters from the input, and built up a baseline-only model (Figure S3f) using the fluorescence in the native baseline (20–50 °C) only.

**Fig. 4:**
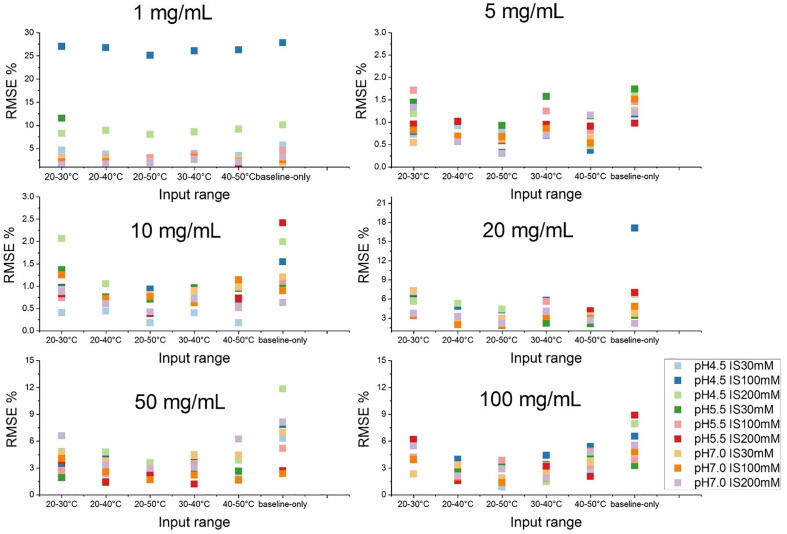
The deviation of the ML model-derived denaturation curves to the experiment data for different data input range to the model. RMSE % is the ratio between the RMSE of fluorescence intensity for 52–90 °C of the experiment and model-predicted values to the fluorescence intensity at the *T*_m_.

The quality of model-predicted curves from different input temperature ranges of the fluorescence was compared with the experimental curves (Fig. 3). The RMSE% shows the model-to-experiment RMSE deviation in fluorescence intensity throughout 52–90 °C relative to the fluorescence intensity of the experimental curve at the midpoint (*T*_m_). Most RMSE% falls below 10% over the concentration, pH and IS, with a number of exceptional datasets below 3% found at 5 and 10 mg/mL, regardless of the range of input data (Fig. 4). RMSE% was the greatest at 1 mg/mL, pH 4.5, IS 100 mM / 200 mM. In general, using the input ranges of 20–50 °C and 40–50 °C gave the lowest RMSE% (in some cases, such as at 50 mg/mL and 100 mg/mL, the RMSE for 20–50 °C was better than for 40–50 °C), indicating that the modelled curves from these input ranges most closely resembled the experimental curves. For each concentration, using only the baseline fluorescence data as the input gave marginally greater deviations from experimental curves on average.

### 3.3 Conformational stability obtained from experimental and model-predicted data

**Fig. 5:**
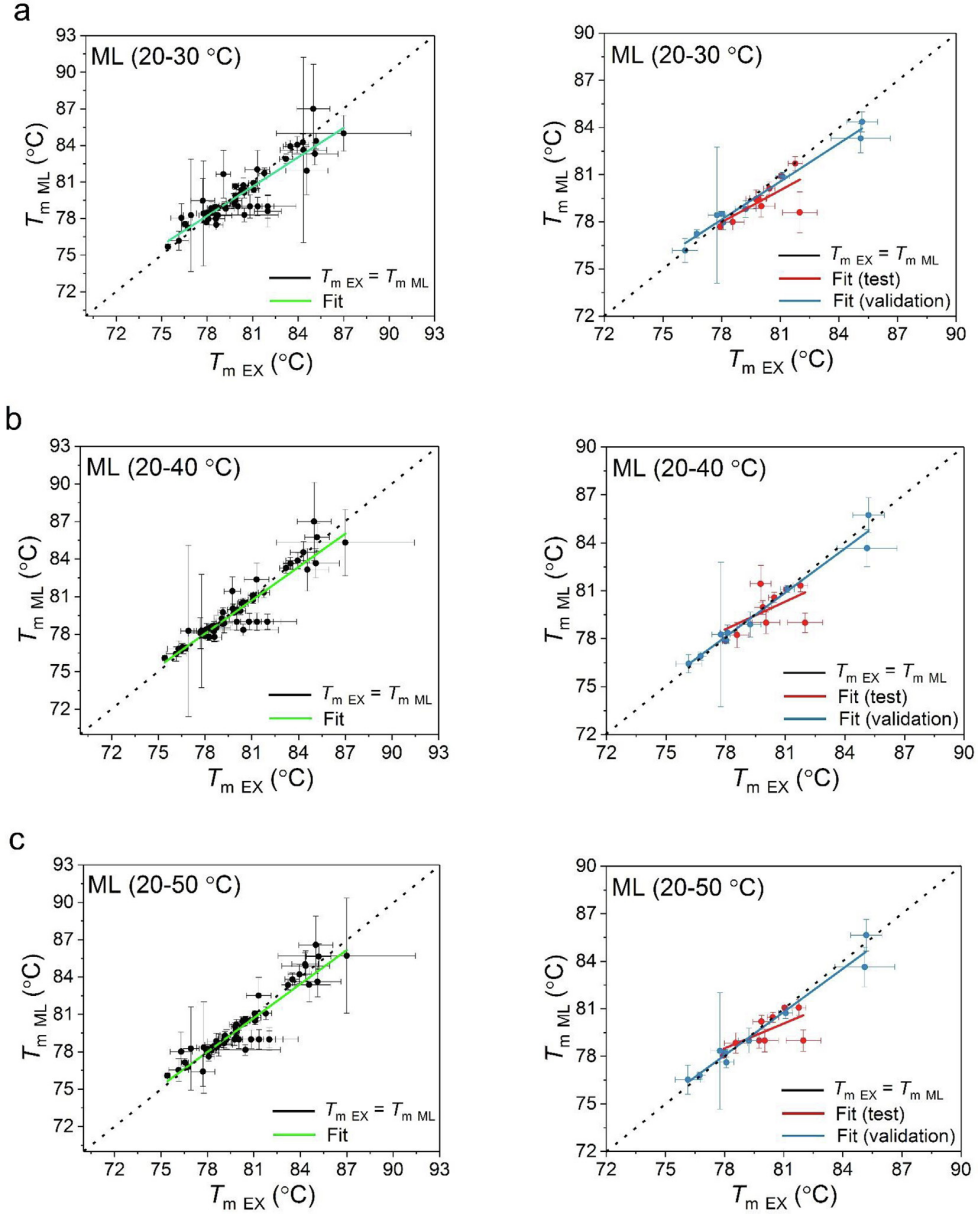

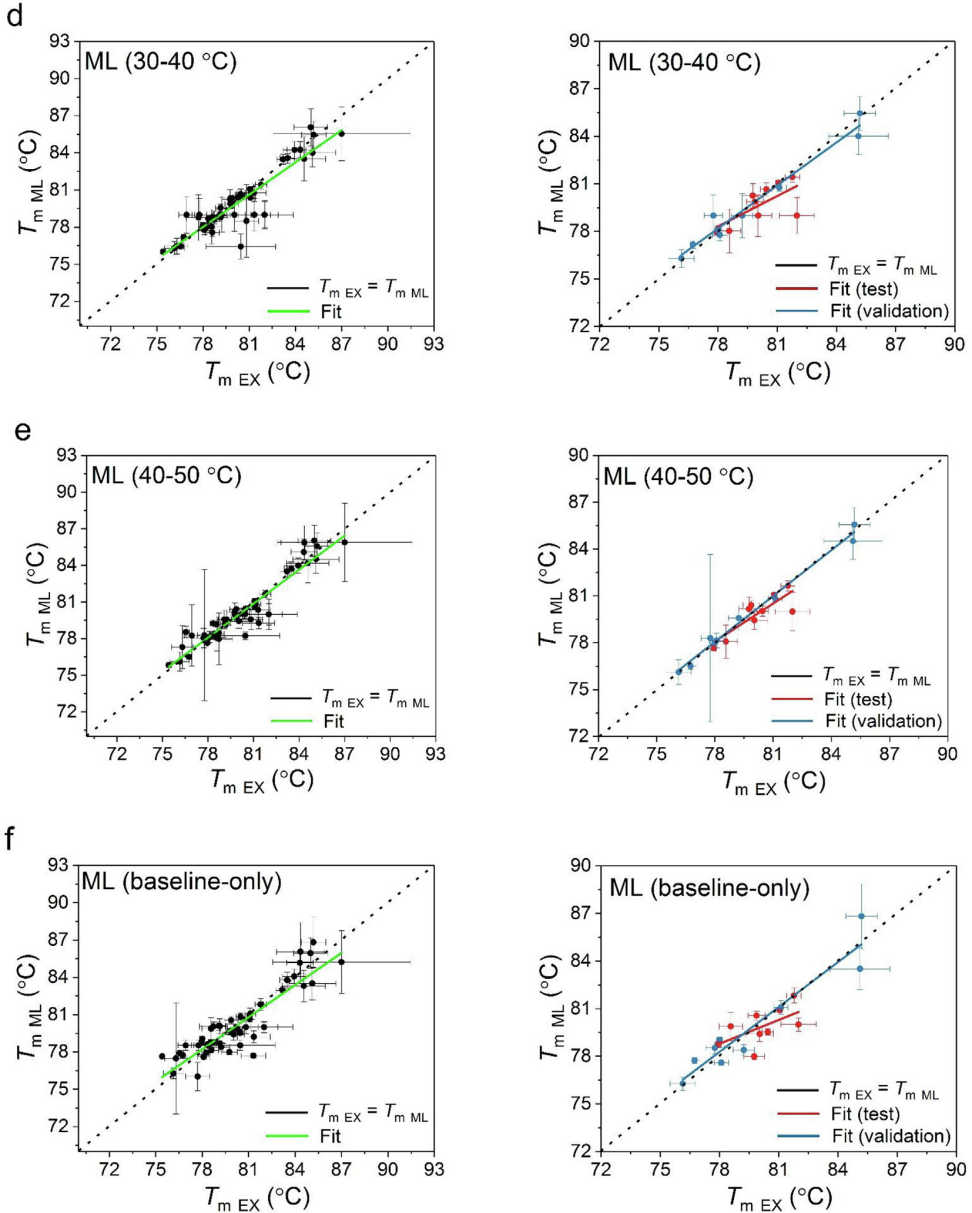
Parity plot of the *T*_m_ obtained from the experimental and ML-derived data. The results are presented based on the temperature range of the experimental fluorescence used to train the ML model (a) 20–30 °C (b), 20–40 °C (c), 20–50 °C (d), 30–40 °C (e), 40–50 °C and (f) the prediction from only the fluorescence data in the native baseline omitting the pH, IS and protein concentration information. The *T*_m_ obtained from the validation and test datasets are plotted and fitted separately. The “ideal” scenario where *T*_m, Ex_ is equal to *T*_m, ML_ (i.e. slope = 1) is shown as black diagonal dash line.

The unfolding transition midpoints (*T*_m_) obtained from the experimental and model-predicted curves show a good consistency over the experimental conditions (Fig. 5 and Table 2). Therefore, the *T*_m_ derived from model-predicted curves are observed to present similar trends of change over the concentration and buffer conditions, as those of obtained directly from the experimental data fitting (Figure S1). However, minor differences were observed from the predictive results when modelling from different input ranges. A better consistency with experimental *T*_m_ was obtained when larger input data volumes were used, as expected. The RMSE values between *T*_m_ values obtained from 20 to 50 °C, 20–40 °C, and 20–30 °C input data were 1.0, 1.0, and 1.2 °C, respectively, for the entire dataset; and 1.1, 1.2, and 1.2 °C, for the test dataset alone (Table 2). Interestingly, the quality of model-predicted results improved when using an input range from a higher temperature window, such that the RMSE values for predictions based on 20–30 °C, 30–40 °C, and 40–50 °C input data, were 1.2, 1.1, and 0.8 °C, for both the entire dataset, and the test dataset alone.

**Table 2:** Goodness of the correlation between the experimental and ML-derived *T*_m_ values. The slope and R^2^ are obtained from the linear fit of the data in Fig. 5. The correlation with experimental data was also compared within each ML group between the validation, test and the entire datasets.

Input range		RMSE (°C)	Slope	R^2^
20–30 °C	All *T*_m_ values	1.21	0.80 ± 0.06	0.80
	*T*_m_ from validation	0.75	0.81 ± 0.45	0.98
	*T*_m_ from test	1.22	0.68 ± 0.23	0.48
20–40 °C	All *T*_m_ values	1.01	0.89 ± 0.05	0.85
	*T*_m_ from validation	0.57	0.92 ± 0.06	0.97
	*T*_m_ from test	1.21	0.57 ± 0.31	0.33
20–50 °C	All *T*_m_ values	1.01	0.91 ± 0.05	0.86
	*T*_m_ from validation	0.61	0.91 ± 0.06	0.97
	*T*_m_ from test	1.12	0.52 ± 0.23	0.42
30–40 °C	All *T*_m_ values	1.10	0.87 ± 0.06	0.83
	*T*_m_ from validation	0.61	0.90 ± 0.06	0.97
	*T*_m_ from test	1.09	0.64 ± 0.26	0.46
40–50 °C	All *T*_m_ values	0.84	0.93 ± 0.04	0.90
	*T*_m_ from validation	0.34	0.98 ± 0.04	0.99
	*T*_m_ from test	0.77	0.81 ± 0.19	0.71
Baseline-only	All *T*_m_ values	1.18	0.86 ± 0.06	0.80
	*T*_m_ from validation	0.99	0.94 ± 0.11	0.91
	*T*_m_ from test	1.12	0.50 ± 0.26	0.35

The linear regression of the test datasets shows larger deviations, compared to those of the validation and the entire datasets. However, the test predictions still fall close to the parity line against experimental values, and so the increased RMSE likely reflects only the relatively small range of test *T*_m_ values between 78 and 82 °C.

## 4 Discussion

The thermal unfolding transition of proteins from which the conformational stability is determined, is usually measured by the intrinsic fluorescence (differential scanning fluorimetry), molecular chirality (circular dichroism), or specific heat capacity (differential scanning calorimetry)[27], [28], [29], [30], [31], [32], [33]. Although the native structure of a protein is largely homogeneous, it is not a rigid body but undergoes continual dynamic motion, including frequent unfolding and conformational switching of local regions of structure [34], [35]. Under the native state, the intrinsic fluorescence of a protein reflects the average local environment around tryptophan, tyrosine or phenylalanine residues for the whole native ensemble of structures. This fluorescence is sensitive to protein conformational changes induced by changes in temperature, protein concentration and the buffer solution conditions [36]. The local unfolding events, conformational states, and protein–protein interactions within the native ensemble directly influence the stability of the protein to global unfolding, and hence probing fluorescence under native conditions has the potential to reveal the thermodynamic propensity to globally unfold.

The conformational stability of an antibody fragment, Fab, was studied for 54 combinations of concentration, pH and ionic strength. The fluorescence intensity of Fab increased as unfolding exposed tryptophan residues to the solvent and eventually decreased as Fab was fully denatured. The resulting denaturation profiles throughout the thermal transition were compared to their model-predicted twin datasets predicted from only the spectra obtained below the transition temperature. The model-predicted curves show similar profiles to their experimental twins and the RMSE% indicated that the discrepancy of the fluorescence signals across the temperature range was less than 10%. This provided a high-quality prediction with the model-predicted curves, for further analysis using the same thermal-unfolding model fitted to the experimental data. The *T*_m_ values obtained from ML models showed a robust agreement with the equivalent experimentally determined values. Altogether, the fluorescence measurement and ML depicted similar behaviours of Fab over changes in protein concentration, acidic to neutral pH, and from low to high ionic strength.

Consistent with previous work, the Fab became apparently self-stabilized at high concentrations [26]. Moreover, *T*_m_ at 100 mg/mL became very similar across the pH and IS range, suggesting some “protective” species formed during the unfolding/aggregation process makes the protein stability resistant to changes in buffer environment. The balance of aggregation and denaturation behaviour clearly changes from the lower to the higher concentrations. This complex shift in the protein denaturation pathway was still successfully modelled by ML, indicating the robustness of the approach, and the ability of ML to unpick different outcomes based on the input spectra.

We attempted to consider which specific features in the native-state fluorescence spectra may have been adopted by the ML algorithm, to predict the unfolding transition. As described above, the prediction improved when using an input range of temperatures closer to the transition region. This was most likely due to a closer correlation between the high-temperature native structure ensemble encoded in the spectra and the states that lead to protein unfolding, as expected due to Hammond effects on the free-energy [37]. However, we could not identify any simple linear relationships between features of the input data and the experimental *T*_m_ values. We fitted the denaturation curves at each wavelength between 330 and 350 nm to investigate the wavelength-dependent change of features (Figure S7 & S8, Table S3). For example, the native baseline slope, and the initial fluorescence intensity at 20 °C, each varied significantly within each buffer condition, due to changes in protein concentration, but with no clear relationship to experimentally-determined *T*_m_ values in most cases (Figure S7 & S8). In addition, the buffer conditions affected the *T*_m_ values, but these did not correlate with the native baseline slope or initial intensity. Furthermore, the experimental *T*_m_ values obtained were also dependent on the wavelength used for denaturation curves within each buffer condition, particularly at the higher protein concentrations, but these were not consistently linked to either the initial fluorescence intensity or the native baseline slopes. Instead, the ML model must have relied upon more complex relationships across these various features. The neural network algorithm could grasp these features together with other unknown ones from the native state baseline to make an overall linkage to the spectroscopic signal in the transition and denatured states.

This study provides a predictive approach that could be used to accelerate the development of stable therapeutic protein formulations. Therapeutic proteins, such as antibody and antibody fragments (full IgG, Fab, scFv, sdAb etc), have become a leading class of pharmaceutical product in recent decades [38], [39], [40], [41]. The conformational stability of a therapeutic protein is important for the safety, efficacy and shelf-life of the product. Any enhancement through optimization of the pharmaceutical formulation can bring a reduction of immunogenicity and an increase in storage stability [42], [42], [43], [44]. Therefore, therapeutic protein candidates under development are usually screened across a wide variety of buffer combinations to identify the most ideal condition to formulate. This approach is highly resource intensive, and uses up protein materials that are often in very short supply at the early stages of development.

The success of the ML predictions show us that the change in thermal stability of a protein in response to different solution conditions, can be entirely predicted from spectra obtained only for the native ensemble. This finding is consistent with the growing understanding that global unfolding events and aggregation mechanisms are critically dependent on local unfolding, conformational states, or protein–protein interactions that occur already within the native ensemble. Therefore, future protein engineering and formulation endeavours can focus on controlling these properties within the native ensemble.

We aim to build this method to accelerate the drug development process by 1) experimentally validating less buffer conditions and 2) only low temperature is needed for screening. Furthermore, the running time of FFNN neural network in this study can process the total 2268 thermal denaturation curves in about 15 min, by obtaining fluorescence spectra in 10 mins over only a 10 °C range of the pre-transition region, which greatly accelerated the processing time compared to conventional data analysis methods which usually measure in 70 mins over a 70 °C range. An additional advantage is that the protein does not need to be denatured, often irreversibly causing aggregation, and would therefore be re-useable for other experiments. This work shows the potential and capability of the incorporation of ML into the future digital platform for the developability characterization of biopharmaceutical products. In future investigations, we plan to test the generality of this method on a wider range of buffer conditions and on other proteins of different sizes, structure, surface charge and stability. We aim to achieve a better robustness *via* training the ML algorithm from a larger volume of datasets spanning from different proteins and protein families.

## 5 Conclusion

In this work, we applied the Feedforward Neural Network model to study the intrinsic fluorescence of the native state of Fab, a multi-domain therapeutic protein, to derive the fluorescence spectra in the transition and denatured state regions of the thermal denaturation profile. The *T*_m_ derived from the experiments and from the predictive model were highly correlated, showing that there is sufficient information in the temperature-dependent native state spectra of proteins, to derive their conformational stability. Based on this discovery, a non-denaturing measurement can be developed to make fast prediction of the stability of a therapeutic protein under different formulation conditions.

## Declaration of Competing Interest

The authors declare that they have no known competing financial interests or personal relationships that could have appeared to influence the work reported in this paper.
