# Supplementary material for: Machine learning reveals hidden stability code in protein native fluorescence
Source: Comput Struct Biotechnol J. 2021 Apr 28;19:2750–60. doi: 10.1016/j.csbj.2021.04.047 (PMC8131987; doi:10.1016/j.csbj.2021.04.047)
Supplement: Supplementary data 1 [file mmc1.pdf]

# Supporting information

# Machine learning reveals hidden stability code in protein native fluorescence

Hongyu Zhang[1,2 *], Yang Yang[1,2 *], Cheng Zhang[1], Suzanne S. Farid[1,2], Paul A. Dalby[1,2**]

1 Department of Biochemical Engineering, UCL, London, UK, WC1E 6BT

2 EPSRC Future Targeted Healthcare Manufacturing Hub, UCL, London, UK, WC1E 6BT

* These authors contributed equally to the manuscript

**Correspondence to p.dalby@ucl.ac.uk

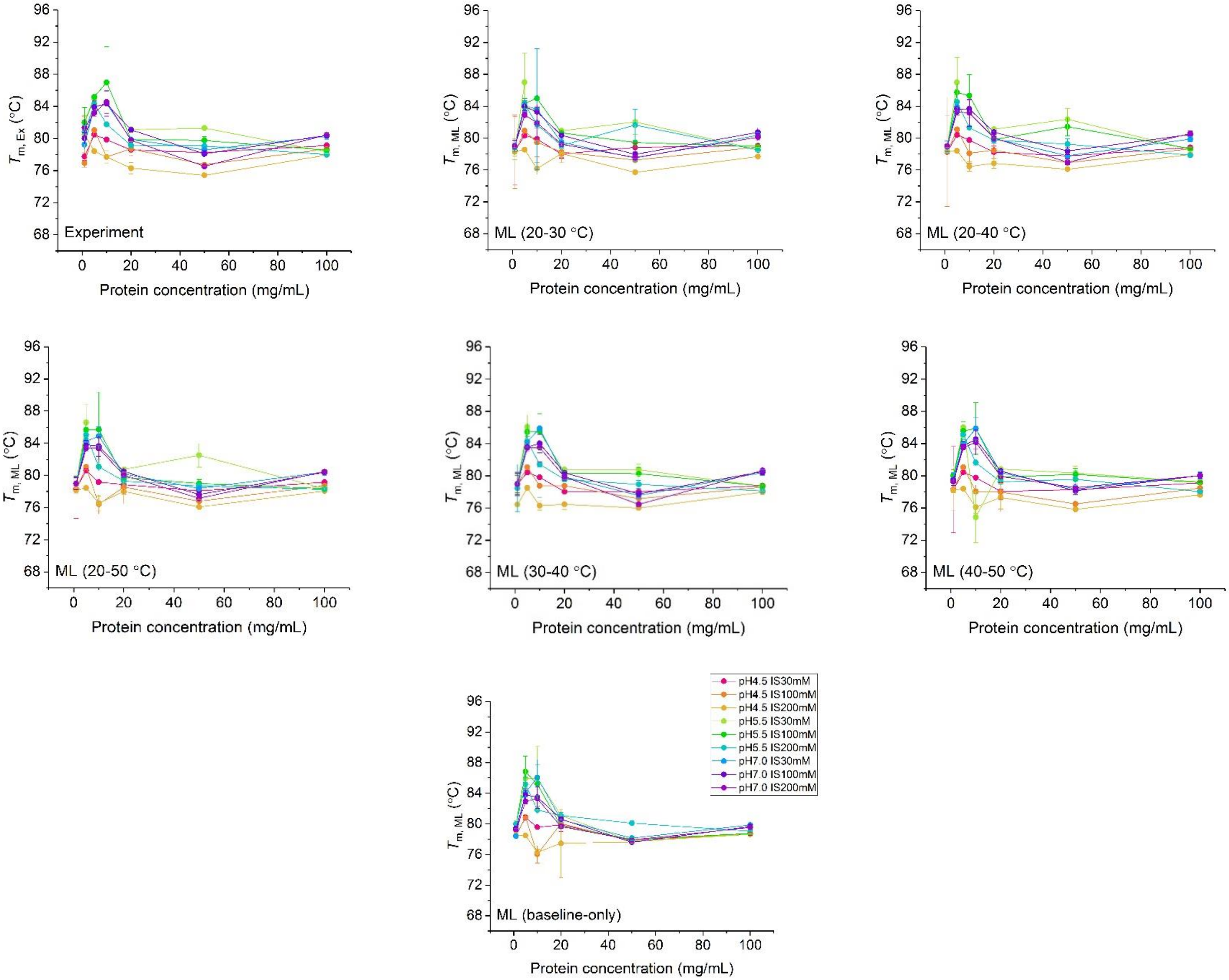


FIGURE S1: $T_m$ from experiment and ML over buffer conditions shows convergence towards 100 mg/mL.

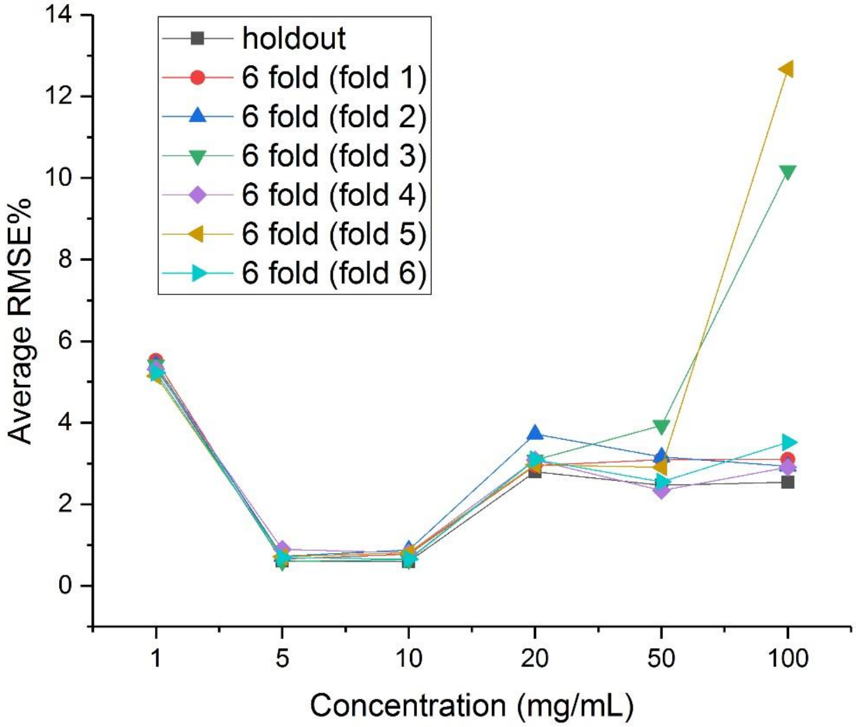


FIGURE S2: Comparison of the performance of the holdout method and 6-fold cross validation as the RMSE% averaged across pH and IS for each protein concentration. To test the robustness of the holdout data splitting method used in this study, a k-fold cross validation method (k=6) was conducted toon the entire 54 datasets, with 20-50 °C data as input. The performance (measured as MSE) of the 6-fold cross-validation had an average MSE of 24,333 compared to 22,043 for the holdout method. The similar performance demonstrated that the splitting strategy applied in the holdout method successfully avoid overfitting or bias issues. The holdout method had the additional benefit of using only 1/6th of the time compared to 6-fold cross validation to, while retaining a similar performance result. The RMSE% of the ML-derived (by holdout method or 6-fold cross validation) curve to the experimental curve was obtained relative to the fluorescence at the midpoint of the experimental curve. The RMSE% was then averaged across the experimental conditions (pH, IS) for each protein concentration (Figure 2c). The results obtained from the holdout method iweres similar to most cross - validation results and sometimes better than cross validation at high protein concentration, demonstrating that data splitting by holdout in this study givesave good robustness.

a

b

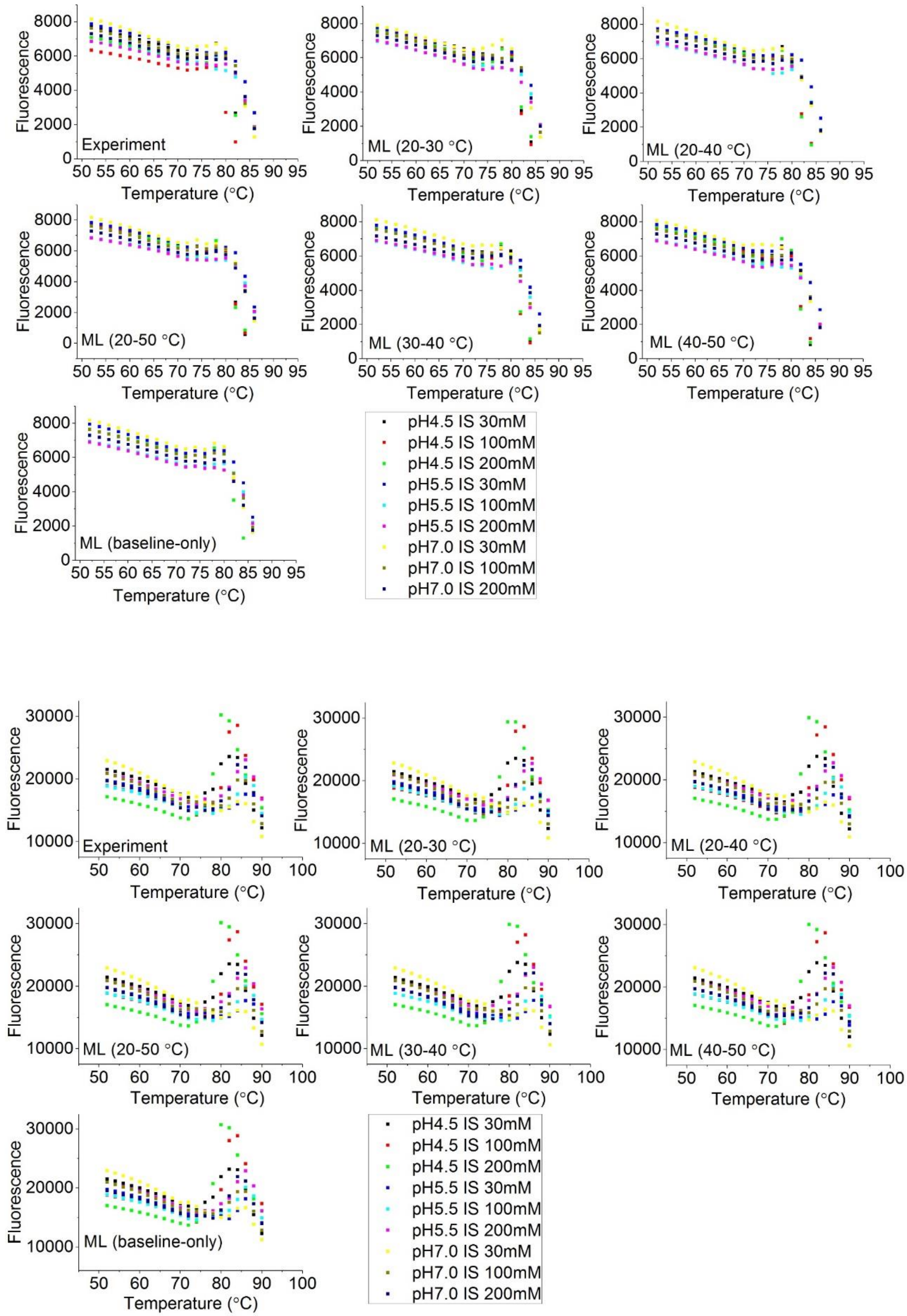
Experiment
ML (20-30 °C)
ML (20-40 °C)
ML (20-50 °C)
ML (30-40 °C)
ML (40-50 °C)
ML (baseline-only)
Fluorescence
Temperature (°C)
pH4.5 IS 30mM
pH4.5 IS 100mM
pH4.5 IS 200mM
pH5.5 IS 30mM
pH5.5 IS 100mM
pH5.5 IS 200mM
pH7.0 IS 30mM
pH7.0 IS 100mM
pH7.0 IS 200mM

c

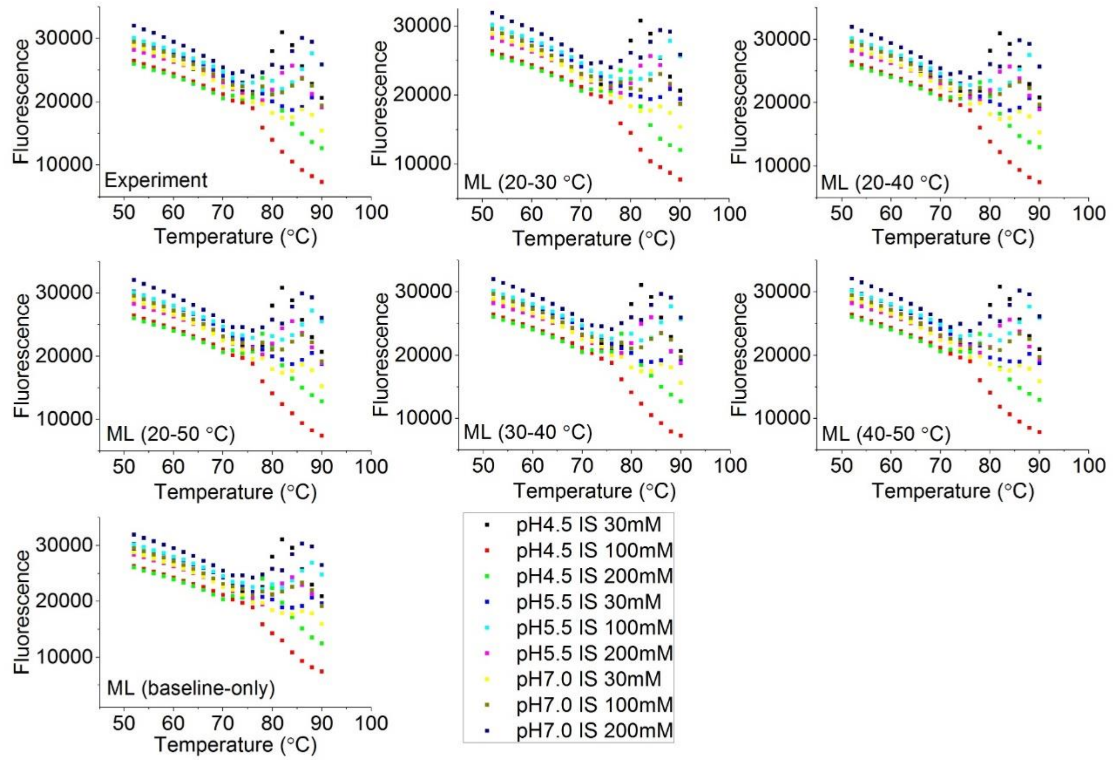
Fluorescence
Temperature (°C)
Experiment
ML (20-30 °C)
ML (20-40 °C)
ML (20-50 °C)
ML (30-40 °C)
ML (40-50 °C)
ML (baseline-only)
pH4.5 IS 30mM
pH4.5 IS 100mM
pH4.5 IS 200mM
pH5.5 IS 30mM
pH5.5 IS 100mM
pH5.5 IS 200mM
pH7.0 IS 30mM
pH7.0 IS 100mM
pH7.0 IS 200mM

d

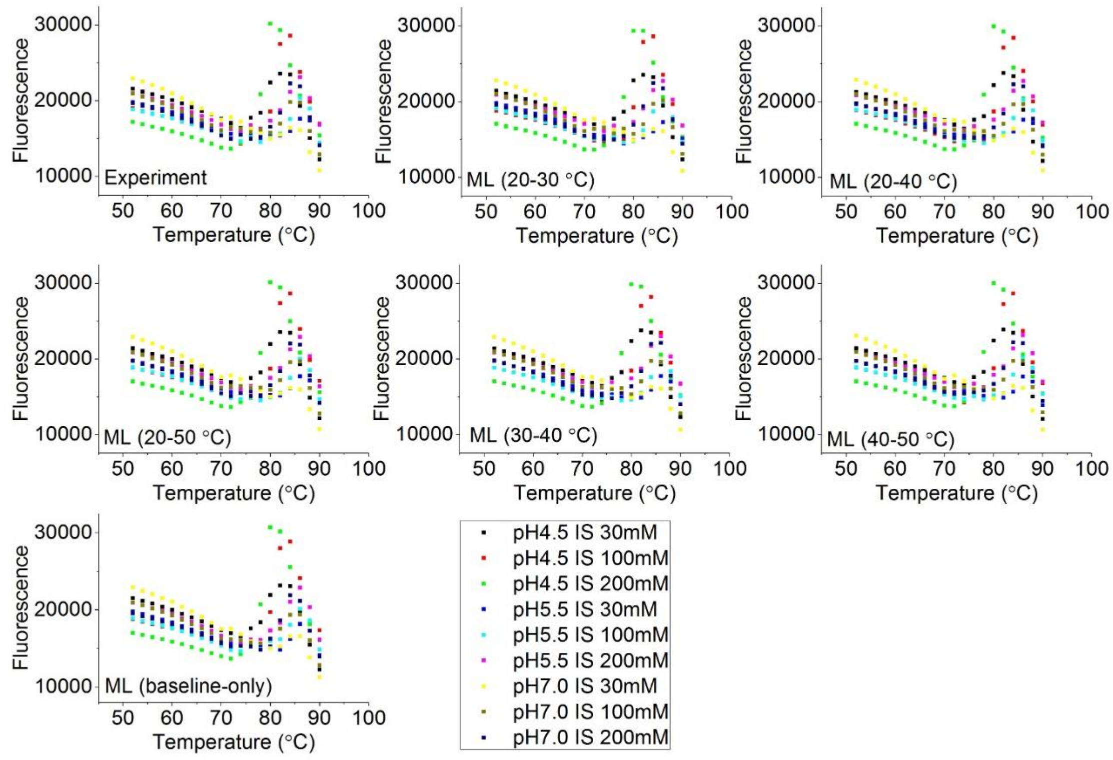
Fluorescence
Temperature (°C)
Experiment
ML (20-30 °C)
ML (20-40 °C)
ML (20-50 °C)
ML (30-40 °C)
ML (40-50 °C)
ML (baseline-only)
pH4.5 IS 30mM
pH4.5 IS 100mM
pH4.5 IS 200mM
pH5.5 IS 30mM
pH5.5 IS 100mM
pH5.5 IS 200mM
pH7.0 IS 30mM
pH7.0 IS 100mM
pH7.0 IS 200mM

e

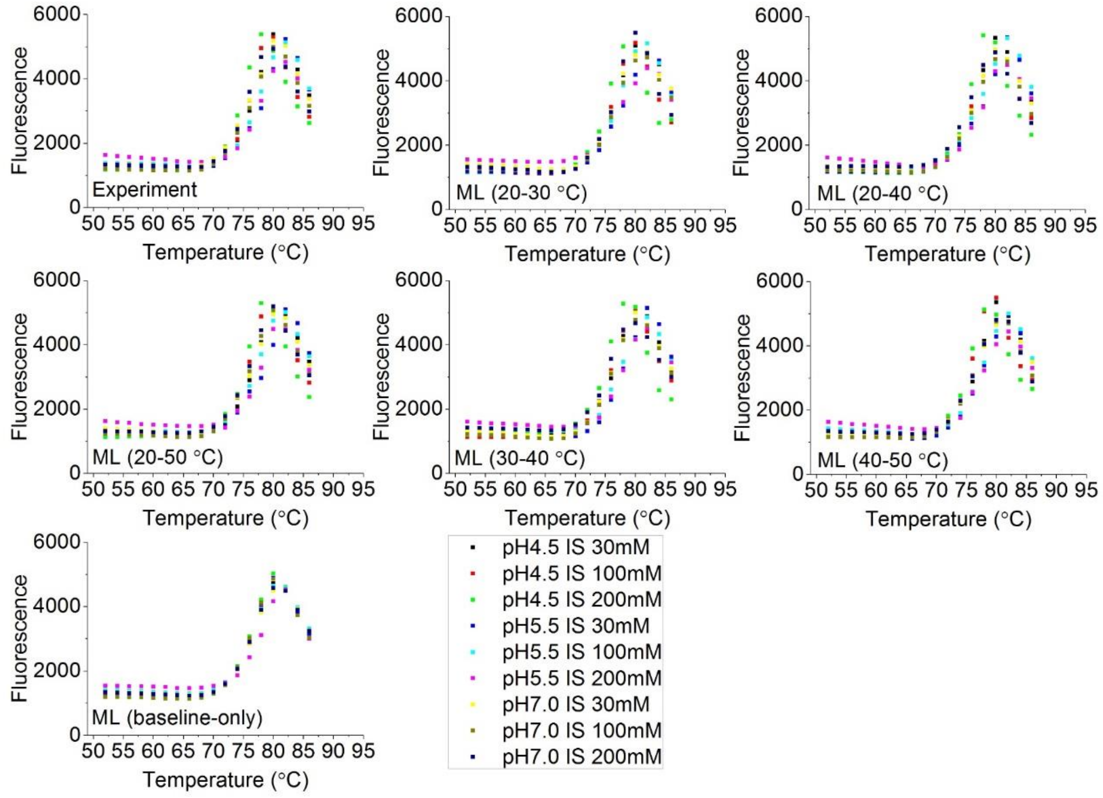
Experiment
ML (20-30 °C)
ML (20-40 °C)
ML (20-50 °C)
ML (30-40 °C)
ML (40-50 °C)
ML (baseline-only)
Fluorescence
Temperature (°C)
pH4.5 IS 30mM
pH4.5 IS 100mM
pH4.5 IS 200mM
pH5.5 IS 30mM
pH5.5 IS 100mM
pH5.5 IS 200mM
pH7.0 IS 30mM
pH7.0 IS 100mM
pH7.0 IS 200mM

f

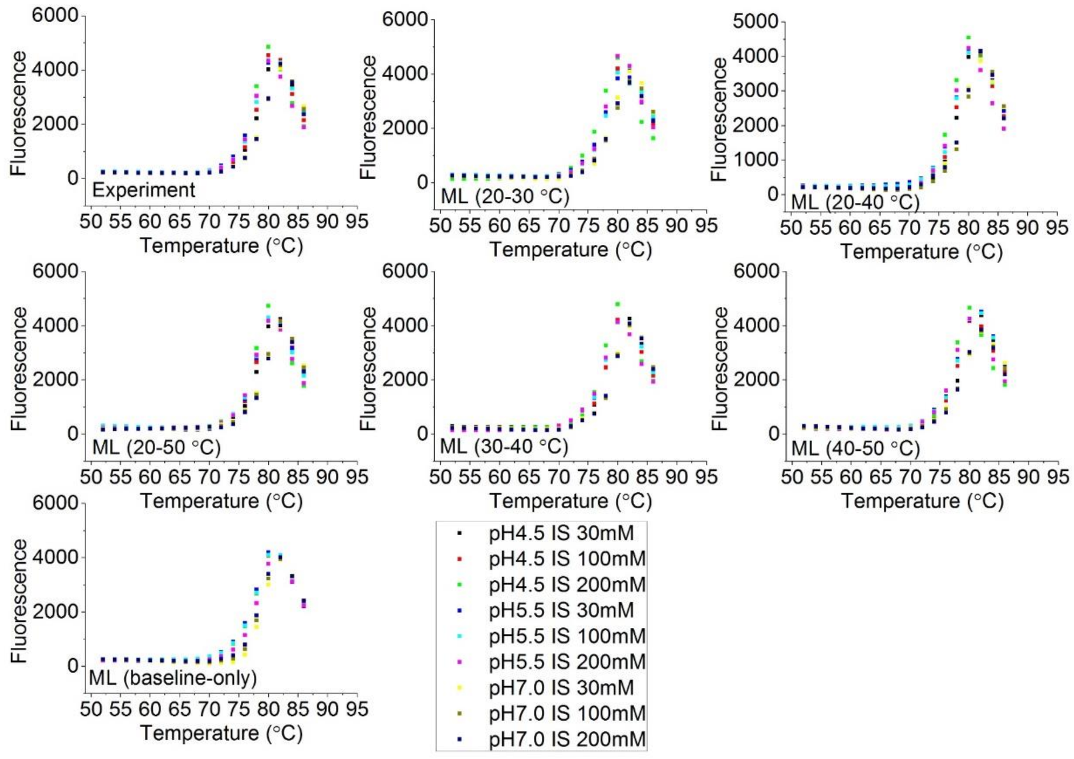
Experiment
ML (20-30 °C)
ML (20-40 °C)
ML (20-50 °C)
ML (30-40 °C)
ML (40-50 °C)
ML (baseline-only)
Fluorescence
Temperature (°C)
pH4.5 IS 30mM
pH4.5 IS 100mM
pH4.5 IS 200mM
pH5.5 IS 30mM
pH5.5 IS 100mM
pH5.5 IS 200mM
pH7.0 IS 30mM
pH7.0 IS 100mM
pH7.0 IS 200mM

FIGURE S3: Comparison between the experimental and ML-derived denaturation curve for the transition region for each concentration: (a) 1 mg/mL, (b) 5 mg/mL, (c) 10 mg/mL, (d) 20 mg/mL, (e) 50 mg/mL and (f) 100 mg/mL.

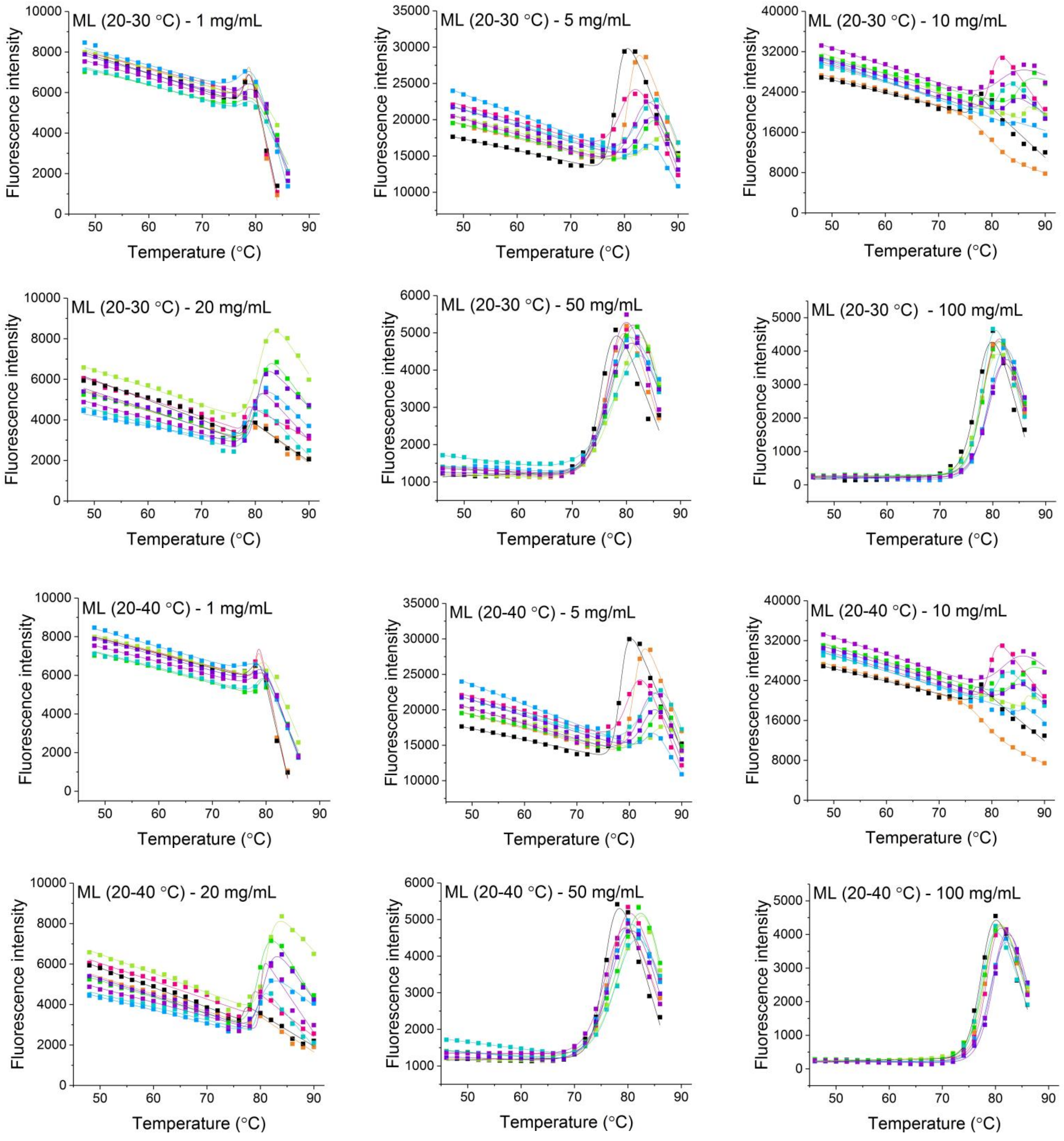
ML (20-30 °C) - 1 mg/mL
ML (20-30 °C) - 5 mg/mL
ML (20-30 °C) - 10 mg/mL
ML (20-30 °C) - 20 mg/mL
ML (20-30 °C) - 50 mg/mL
ML (20-30 °C) - 100 mg/mL
ML (20-40 °C) - 1 mg/mL
ML (20-40 °C) - 5 mg/mL
ML (20-40 °C) - 10 mg/mL
ML (20-40 °C) - 20 mg/mL
ML (20-40 °C) - 50 mg/mL
ML (20-40 °C) - 100 mg/mL
Fluorescence intensity
Temperature (°C)

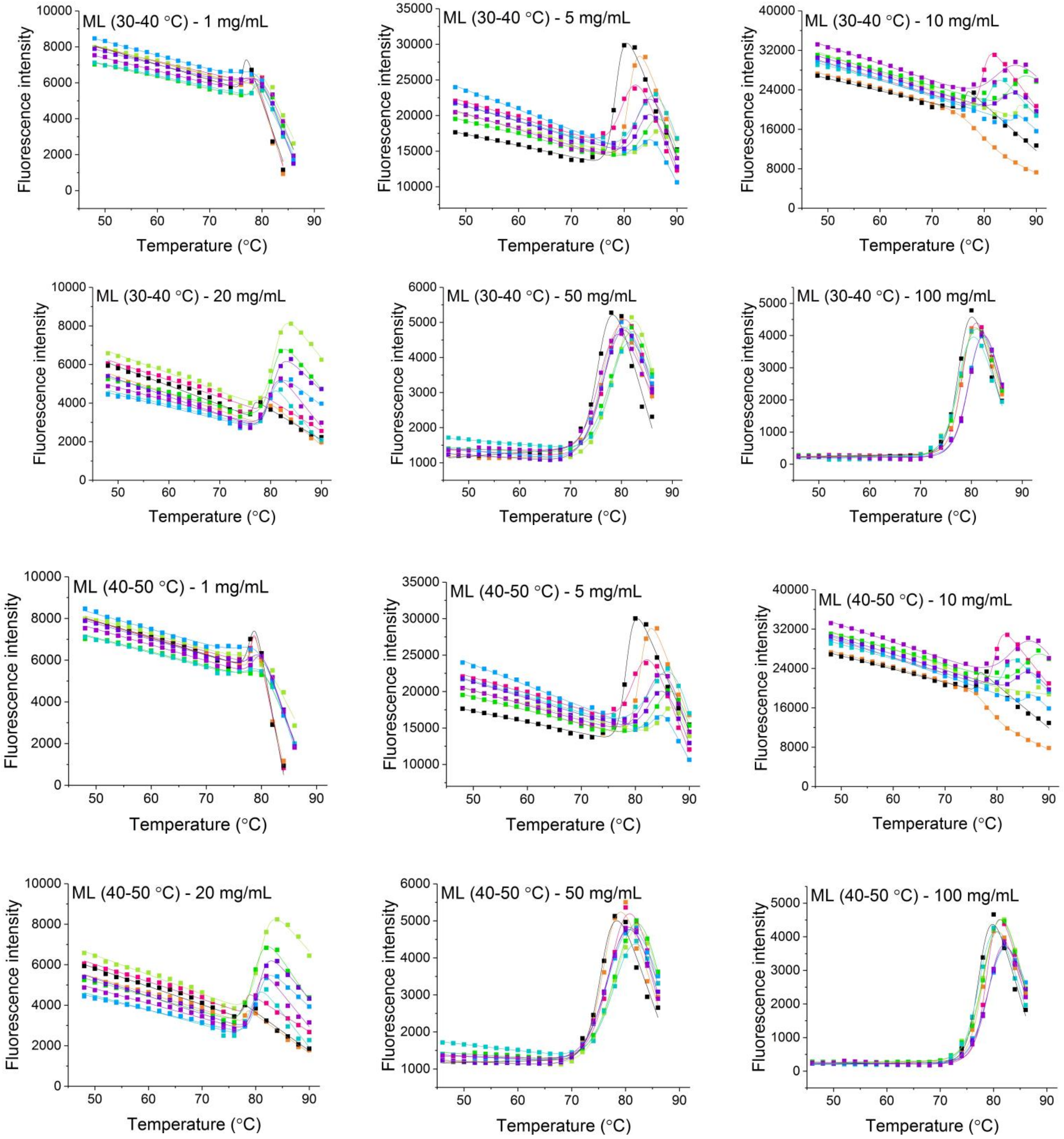
ML (30-40 °C) - 1 mg/mL
ML (30-40 °C) - 5 mg/mL
ML (30-40 °C) - 10 mg/mL
ML (30-40 °C) - 20 mg/mL
ML (30-40 °C) - 50 mg/mL
ML (30-40 °C) - 100 mg/mL
ML (40-50 °C) - 1 mg/mL
ML (40-50 °C) - 5 mg/mL
ML (40-50 °C) - 10 mg/mL
ML (40-50 °C) - 20 mg/mL
ML (40-50 °C) - 50 mg/mL
ML (40-50 °C) - 100 mg/mL
Fluorescence intensity
Temperature (°C)

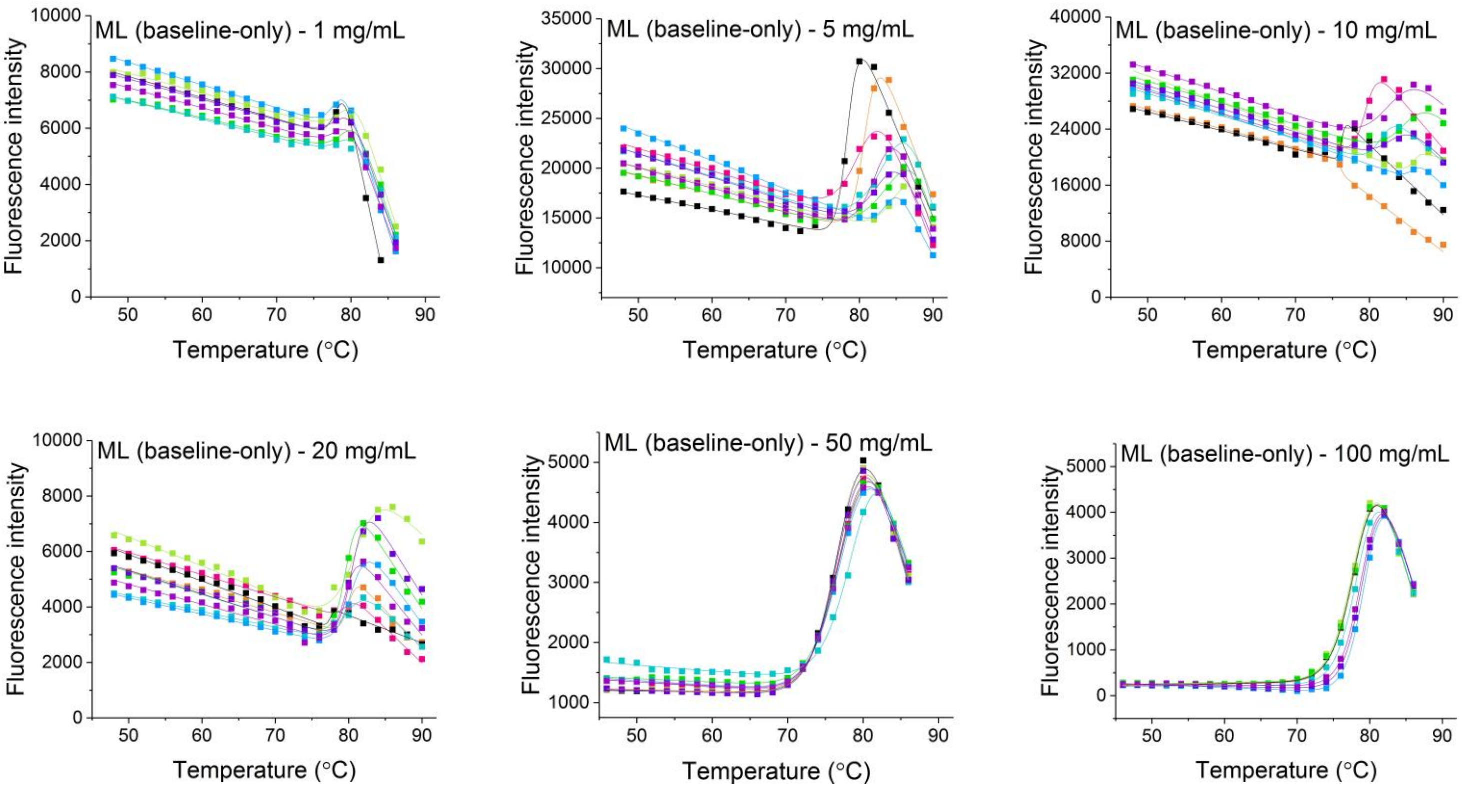


FIGURE S4: Fitting of the ML-derived denaturation curves to the two-state unfolding model. The change in fluorescence intensity at 340 nm was plotted as filled squares against temperature. The data in each buffer is in rainbow colours (pH4.5 IS 30mM: ■, pH4.5 IS 100mM: ■, pH4.5 IS 200mM: ■, pH5.5 IS 30mM: ■, pH5.5 IS 100mM: ■, pH5.5 IS 200mM: ■, pH7.0 IS 30mM: ■, pH7.0 IS 100mM: ■, pH7.0 IS 200mM: ■).

| Buffer condition | $T_{m,\ Ex}$ (°C) |
| --- | --- |

| pH | IS (mM) | | 1 mg/mL | 5 mg/mL | 10 mg/mL | 20 mg/mL | 50 mg/mL | 100 mg/mL |
|---|---|---|---|---|---|---|---|---|
| 4.5 | 30 | | 77.8 ± 0.5 | 80.5 ± 0.3 | 79.9 ± 0.2 | 78.6 ± 0.6 | 78.3 ± 0.3 | 79.1 ± 0.2 |
| | 100 | | 76.9 ± 0.5 | 81.1 ± 0.1 | 77.7 ± 0.8 | 78.7 ± 0.9 | 76.7 ± 0.2 | 78.7 ± 0.1 |
| | 200 | | 80.5 ± 2.0 | 78.4 ± 0.1 | 76.1 ± 0.7 | 76.3 ± 0.7 | 75.4 ± 0.1 | 78.0 ± 0.1 |
| 5.5 | 30 | | 82.0 ± 0.9 | 85.0 ± 1.1 | N.D | 81.1 ± 0.4 | 81.3 ± 0.8 | 78.4 ± 0.2 |
| | 100 | | 82.0 ± 1.9 | 85.2 ± 0.8 | 87.0 ± 4.4 | 79.9 ± 0.2 | 79.8 ± 0.5 | 78.6 ± 0.2 |
| | 200 | | 80.8 ± 0.8 | 84.3 ± 0.9 | 81.8 ± 0.4 | 79.2 ± 0.3 | 79.1 ± 0.5 | 78.0 ± 0.2 |
| 7.0 | 30 | | 79.2 ± 0.5 | 84.0 ± 0.8 | 84.4 ± 1.5 | 79.9 ± 0.4 | 78.6 ± 0.5 | 80.3 ± 0.3 |
| | 100 | | 80.0 ± 0.7 | 83.5 ± 0.4 | 85.1 ± 1.5 | 81.1 ± 0.3 | 78.1 ± 0.4 | 80.4 ± 0.3 |
| | 200 | | 81.3 ± 1.1 | 83.2 ± 0.4 | 84.6 ± 1.4 | 79.8 ± 0.3 | 76.5 ± 0.2 | 80.4 ± 0.3 |
| Buffer condition | | | $T_{m, ML}$ (°C) | | | | | |
| pH | IS (mM) | Prediction range | 1 mg/mL | 5 mg/mL | 10 mg/mL | 20 mg/mL | 50 mg/mL | 100 mg/mL |
| 4.5 | 30 | 20-30°C | 78.4 ± 4.3 | 80.3 ± 0.3 | 79.9 ± 0.2 | 78.0 ± 0.5 | 78.8 ± 0.5 | 79.0 ± 0.2 |
| | | 30-40°C | 79.0 ± 1.3 | 80.4 ± 0.2 | 79.8 ± 0.2 | 78.0 ± 1.4 | 78.0 ± 0.3 | 78.8 ± 0.2 |
| | | 40-50°C | 78.3 ± 5.4 | 80.5 ± 0.2 | 79.8 ± 0.2 | 78.1 ± 1.1 | 78.3 ± 0.3 | 79.1 ± 0.1 |
| | | 20-50°C | 78.3 ± 3.7 | 80.6 ± 0.2 | 79.2 ± 0.3 | 78.9 ± 0.6 | 78.1 ± 0.3 | 79.2 ± 0.2 |
| | | 20-40°C | 78.3 ± 4.5 | 80.4 ± 0.2 | 79.7 ± 0.2 | 78.2 ± 0.8 | 77.8 ± 0.3 | 78.8 ± 0.2 |
| | | Baseline-only | 78.5 ± 0.4 | 80.8 ± 0.3 | 79.6 ± 0.2 | 79.9 ± 0.9 | 77.9 ± 0.2 | 78.7 ± 0.2 |
| | 100 | 20-30°C | 78.3 ± 4.6 | 80.9 ± 0.1 | 79.5 ± 1.8 | 78.3 ± 0.7 | 77.2 ± 0.3 | 78.9 ± 0.2 |
| | | 30-40°C | 78.3 ± 2.5 | 81.1 ± 0.1 | 78.8 ± 1.9 | 78.8 ± 0.5 | 77.2 ± 0.3 | 78.6 ± 0.2 |
| | | 40-50°C | 78.5 ± 0.4 | 81.1 ± 0.1 | 78.1 ± 0.8 | 78.0 ± 2.1 | 76.5 ± 0.2 | 78.5 ± 0.2 |
| | | 20-50°C | 78.3 ± 3.4 | 81.1 ± 0.1 | 76.4 ± 1.2 | 78.6 ± 0.9 | 76.8 ± 0.2 | 78.7 ± 0.2 |
| | | 20-40°C | 78.3 ± 6.8 | 81.1 ± 0.1 | 78.1 ± 1.2 | 78.5 ± 1.0 | 76.9 ± 0.3 | 78.7 ± 0.2 |
| | | Baseline-only | 78.5 ± 0.4 | 80.9 ± 0.1 | 76.0 ± 1.1 | 80.0 ± 0.4 | 77.7 ± 0.2 | 78.8 ± 0.2 |
| | 200 | 20-30°C | 78.3 ± 0.6 | 78.5 ± 0.1 | 76.2 ± 0.8 | 78.1 ± 1.2 | 75.7 ± 0.2 | 77.7 ± 0.2 |
| | | 30-40°C | 76.4 ± 1.0 | 78.5 ± 0.1 | 76.3 ± 0.6 | 76.5 ± 0.6 | 76.0 ± 0.2 | 78.0 ± 0.2 |

| | | | | | | | | |
|---|---|---|---|---|---|---|---|---|
| | | 40-50°C | 78.2 ± 0.3 | 78.4 ± 0.1 | 76.1 ± 0.8 | 77.3 ± 1.8 | 75.8 ± 0.2 | 77.7 ± 0.1 |
| | | 20-50°C | 78.2 ± 0.4 | 78.4 ± 0.1 | 76.5 ± 0.9 | 78.0 ± 1.5 | 76.1 ± 0.2 | 77.9 ± 0.1 |
| | | 20-40°C | 78.3 ± 0.5 | 78.4 ± 0.1 | 76.4 ± 0.6 | 76.8 ± 0.6 | 76.1 ± 0.1 | 78.1 ± 0.2 |
| | | Baseline-only | 78.5 ± 0.4 | 78.5 ± 0.1 | 76.3 ± 0.4 | 77.5 ± 4.5 | 77.7 ± 0.2 | 78.8 ± 0.2 |
| 5.5 | 30 | 20-30°C | 78.6 ± 1.3 | 87.0 ± 3.7 | N.D | 80.9 ± 0.2 | 82.0 ± 1.6 | 78.6 ± 0.3 |
| | | 30-40°C | 79.0 ± 1.1 | 86.1 ± 1.5 | N.D | 80.8 ± 0.2 | 80.8 ± 0.7 | 78.7 ± 0.3 |
| | | 40-50°C | 80.0 ± 1.2 | 86.0 ± 1.3 | 74.9 ± 3.1 | 80.8 ± 0.2 | 80.3 ± 0.9 | 79.2 ± 0.3 |
| | | 20-50°C | 79.0 ± 0.7 | 86.6 ± 2.3 | N.D | 80.7 ± 0.3 | 82.5 ± 1.5 | 78.4 ± 0.2 |
| | | 20-40°C | 79.0 ± 0.6 | 87.0 ± 3.1 | N.D | 81.1 ± 0.2 | 82.4 ± 1.3 | 78.6 ± 0.2 |
| | | Baseline-only | 80.0 ± 0.4 | 85.9 ± 1.2 | 86.0 ± 4.2 | 81.1 ± 0.4 | 77.7 ± 0.2 | 78.6 ± 0.2 |
| | 100 | 20-30°C | 79.0 ± 1.0 | 84.4 ± 0.6 | 85.0 ± 1.5 | 80.7 ± 0.2 | 79.5 ± 0.6 | 79.0 ± 0.2 |
| | | 30-40°C | 79.0 ± 1.1 | 85.4 ± 1.1 | 85.6 ± 2.2 | 80.4 ± 0.2 | 80.3 ± 0.8 | 78.8 ± 0.3 |
| | | 40-50°C | 80.0 ± 0.9 | 85.6 ± 1.1 | 85.9 ± 3.2 | 79.8 ± 0.2 | 80.2 ± 0.8 | 79.2 ± 0.2 |
| | | 20-50°C | 79.0 ± 0.9 | 85.7 ± 1.0 | 85.7 ± 4.6 | 79.7 ± 0.2 | 79.0 ± 0.5 | 78.4 ± 0.2 |
| | | 20-40°C | 79.0 ± 0.6 | 85.7 ± 1.1 | 85.3 ± 2.7 | 79.8 ± 0.2 | 81.4 ± 1.2 | 78.7 ± 0.2 |
| | | Baseline-only | 80.0 ± 0.5 | 86.8 ± 2.0 | 85.2 ± 2.5 | 79.7 ± 1.3 | 78.0 ± 0.2 | 78.8 ± 0.2 |
| | 200 | 20-30°C | 79.0 ± 1.0 | 84.3 ± 0.7 | 81.7 ± 0.5 | 79.1 ± 0.3 | 81.7 ± 2.0 | 78.5 ± 0.2 |
| | | 30-40°C | 78.5 ± 2.9 | 84.3 ± 0.6 | 81.4 ± 0.3 | 79.6 ± 0.3 | 79.0 ± 0.5 | 78.1 ± 0.2 |
| | | 40-50°C | 79.6 ± 0.8 | 85.1 ± 1.0 | 81.7 ± 0.3 | 79.2 ± 0.3 | 79.6 ± 0.8 | 78.0 ± 0.2 |
| | | 20-50°C | 79.0 ± 0.8 | 85.0 ± 0.9 | 81.1 ± 0.5 | 79.3 ± 0.3 | 78.7 ± 0.4 | 78.3 ± 0.2 |
| | | 20-40°C | 79.0 ± 0.7 | 84.5 ± 0.9 | 81.3 ± 0.4 | 79.7 ± 0.4 | 79.2 ± 0.7 | 77.9 ± 0.2 |
| | | Baseline-only | 80.0 ± 0.5 | 85.2 ± 0.9 | 81.8 ± 0.5 | 80.1 ± 0.6 | 80.1 ± 0.3 | 79.1 ± 0.2 |
| 7.0 | 30 | 20-30°C | 78.8 ± 0.6 | 84.1 ± 0.7 | 83.6 ± 7.6 | 79.5 ± 0.4 | 77.5 ± 0.4 | 80.4 ± 0.4 |
| | | 30-40°C | 79.0 ± 1.4 | 84.2 ± 0.7 | 85.9 ± 8.5 | 79.7 ± 0.3 | 77.6 ± 0.3 | 80.4 ± 0.4 |
| | | 40-50°C | 79.6 ± 0.8 | 84.0 ± 0.6 | 85.9 ± 1.4 | 80.4 ± 0.3 | 78.4 ± 0.6 | 80.1 ± 0.5 |
| | | 20-50°C | 79.0 ± 0.8 | 84.2 ± 0.8 | 84.9 ± 1.2 | 80.2 ± 0.4 | 78.4 ± 0.5 | 80.4 ± 0.4 |

| | | | | | | | | |
|---|---|---|---|---|---|---|---|---|
| | | 20-40°C | 78.9 ± 0.8 | 83.9 ± 0.6 | N.D | 80.0 ± 0.4 | 77.8 ± 0.4 | 79.9 ± 0.3 |
| | | Baseline-only | 78.4 ± 0.3 | 84.1 ± 0.6 | 86.1 ± 2.3 | 80.6 ± 0.3 | 78.1 ± 0.2 | 79.9 ± 0.2 |
| | 100 | 20-30°C | 79.0 ± 1.3 | 83.9 ± 0.5 | 83.3 ± 0.9 | 80.4 ± 0.2 | 78.0 ± 0.5 | 80.7 ± 0.6 |
| | | 30-40°C | 79.3 ± 0.7 | 83.6 ± 0.4 | 84.0 ± 1.1 | 80.4 ± 0.3 | 77.8 ± 0.4 | 80.5 ± 0.4 |
| | | 40-50°C | 79.4 ± 0.6 | 83.8 ± 0.5 | 84.5 ± 1.2 | 80.6 ± 0.2 | 78.1 ± 0.5 | 80.0 ± 0.4 |
| | | 20-50°C | 79.0 ± 0.7 | 83.8 ± 0.5 | 83.6 ± 1.2 | 80.5 ± 0.3 | 77.6 ± 0.4 | 80.4 ± 0.3 |
| | | 20-40°C | 79.0 ± 0.7 | 83.7 ± 0.5 | 83.7 ± 1.2 | 80.7 ± 0.3 | 78.4 ± 0.5 | 80.5 ± 0.3 |
| | | Baseline-only | 79.4 ± 0.5 | 83.8 ± 0.6 | 83.5 ± 1.3 | 80.6 ± 0.2 | 77.6 ± 0.2 | 79.7 ± 0.2 |
| | 200 | 20-30°C | 79.0 ± 0.7 | 82.9 ± 0.4 | 81.9 ± 2.0 | 79.2 ± 0.3 | 77.6 ± 0.2 | 80.1 ± 0.4 |
| | | 30-40°C | 79.0 ± 1.3 | 83.5 ± 0.4 | 83.5 ± 1.8 | 79.9 ± 0.3 | 76.5 ± 0.3 | 80.7 ± 0.4 |
| | | 40-50°C | 79.3 ± 0.5 | 83.5 ± 0.5 | 84.2 ± 1.6 | 79.9 ± 0.2 | 78.5 ± 0.5 | 80.0 ± 0.3 |
| | | 20-50°C | 79.0 ± 0.8 | 83.4 ± 0.4 | 83.4 ± 1.4 | 80.0 ± 0.3 | 77.2 ± 0.3 | 80.5 ± 0.3 |
| | | 20-40°C | 79.0 ± 0.7 | 83.3 ± 0.4 | 83.2 ± 1.7 | 80.0 ± 0.4 | 77.0 ± 0.5 | 80.6 ± 0.3 |
| | | Baseline-only | 79.2 ± 0.5 | 83.0 ± 0.3 | 83.3 ± 1.3 | 79.7 ± 0.3 | 77.9 ± 0.2 | 79.5 ± 0.2 |

TABLE S1: Unfolding midpoint ($T_m$) of Fab obtained from experimental and ML-derived data. The $T_m$ quoted in this table is shown as conversion from absolute temperature (K) to Celsius degree (°C).

| Input | Input range 1 (20-50°C) | Input range 2 (20-40°C) | Input range 3 (20-30°C) | Input range 4 (30-40°C) | Input range 5 (40-50°C) | Input range 6 (Baseline-only) |
|---|---|---|---|---|---|---|
| 1 | Concentration | Concentration | Concentration | Concentration | Concentration | FI at 20 °C |
| 2 | pH | pH | pH | pH | pH | FI at 22 °C |
| 3 | IS | IS | IS | IS | IS | FI at 24 °C |
| 4 | Wavelength | Wavelength | Wavelength | Wavelength | Wavelength | FI at 26 °C |
| 5 | FI at 20 °C | FI at 20 °C | FI at 20 °C | FI at 30 °C | FI at 40 °C | FI at 28 °C |
| 6 | FI at 22 °C | FI at 22 °C | FI at 22 °C | FI at 32 °C | FI at 42 °C | FI at 30 °C |
| 7 | FI at 24 °C | FI at 24 °C | FI at 24 °C | FI at 34 °C | FI at 44 °C | FI at 32 °C |
| 8 | FI at 26 °C | FI at 26 °C | FI at 26 °C | FI at 36 °C | FI at 46 °C | FI at 34 °C |
| 9 | FI at 28 °C | FI at 28 °C | FI at 28 °C | FI at 38 °C | FI at 48 °C | FI at 36 °C |
| 10 | FI at 30 °C | FI at 30 °C | FI at 30 °C | FI at 40 °C | FI at 50 °C | FI at 38 °C |
| 11 | FI at 32 °C | FI at 32 °C | N.A | N.A | N.A | FI at 40 °C |
| 12 | FI at 34 °C | FI at 34 °C | | | | FI at 42 °C |
| 13 | FI at 36 °C | FI at 36 °C | | | | FI at 44 °C |
| 14 | FI at 38 °C | FI at 38 °C | | | | FI at 46 °C |
| 15 | FI at 40 °C | FI at 40 °C | | | | FI at 48 °C |
| 16 | FI at 42 °C | N.A | | | | FI at 50 °C |
| 17 | FI at 44 °C | | | | | N.A |
| 18 | FI at 46 °C | | | | | |
| 19 | FI at 48 °C | | | | | |
| 20 | FI at 50 °C | | | | | |
| Output | | | | | | |
| 1 | FI at 52 °C | | | | | |
| 2 | FI at 54 °C | | | | | |
| 3 | FI at 56 °C | | | | | |
| 4 | FI at 58 °C | | | | | |
| 5 | FI at 60 °C | | | | | |
| 6 | FI at 62 °C | | | | | |
| 7 | FI at 64 °C | | | | | |
| 8 | FI at 66 °C | | | | | |
| 9 | FI at 68 °C | | | | | |
| 10 | FI at 70 °C | | | | | |
| 11 | FI at 72 °C | | | | | |
| 12 | FI at 74 °C | | | | | |
| 13 | FI at 76 °C | | | | | |
| 14 | FI at 78 °C | | | | | |
| 15 | FI at 80 °C | | | | | |
| 16 | FI at 82 °C | | | | | |
| 17 | FI at 84 °C | | | | | |
| 18 | FI at 86 °C | | | | | |

| | |
|---|---|
| 19 | FI at 88 °C |
| 20 | FI at 90 °C |

TABLE S2: Data in the neurons of the input and output layer for different ML training model. FI: fluorescence intensity; N.A: not applicable

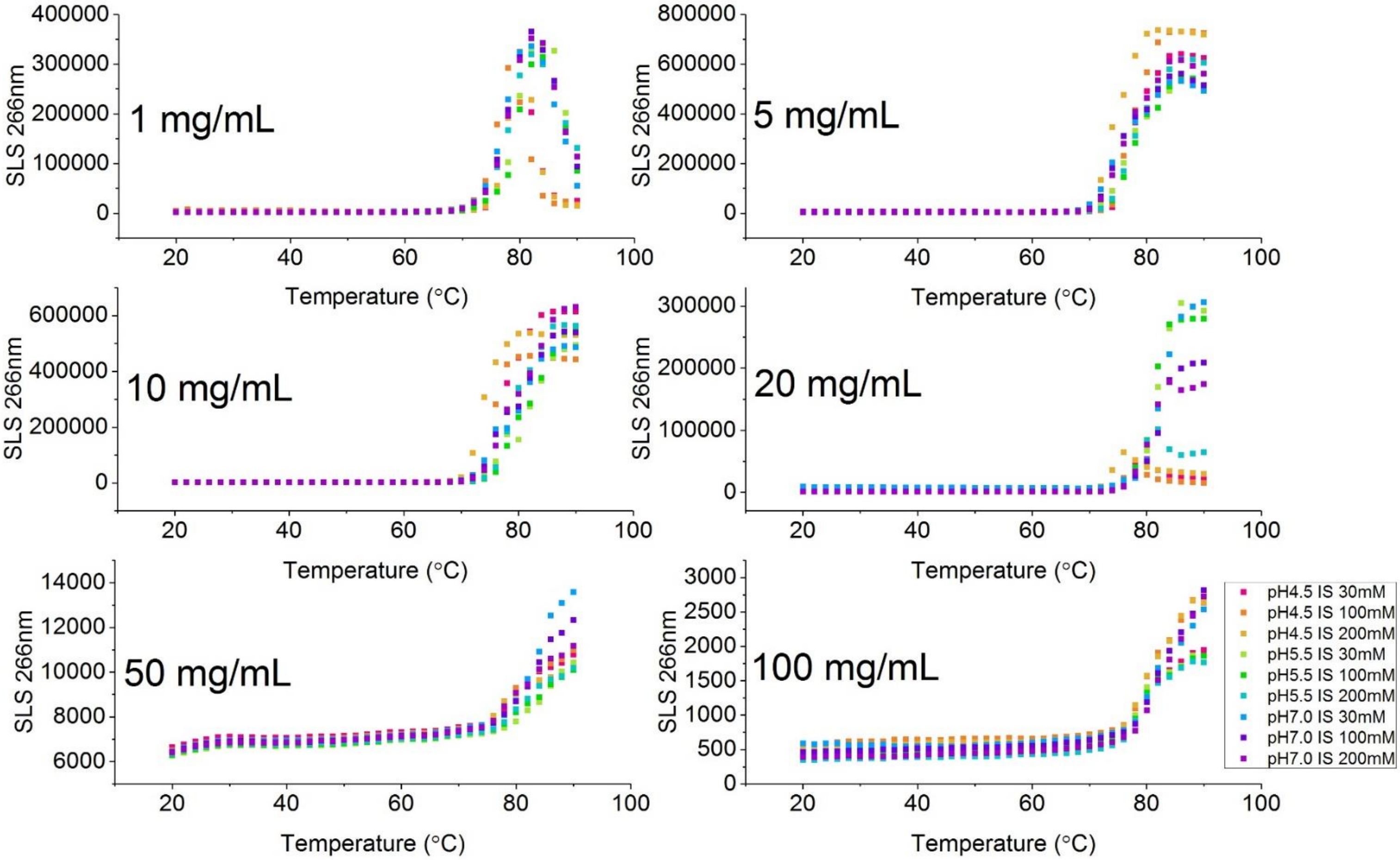


FIGURE S5: Increase in the small aggregate (reported by SLS at 266 nm) with increase in temperature for Fab from 1 to 100 mg/mL.

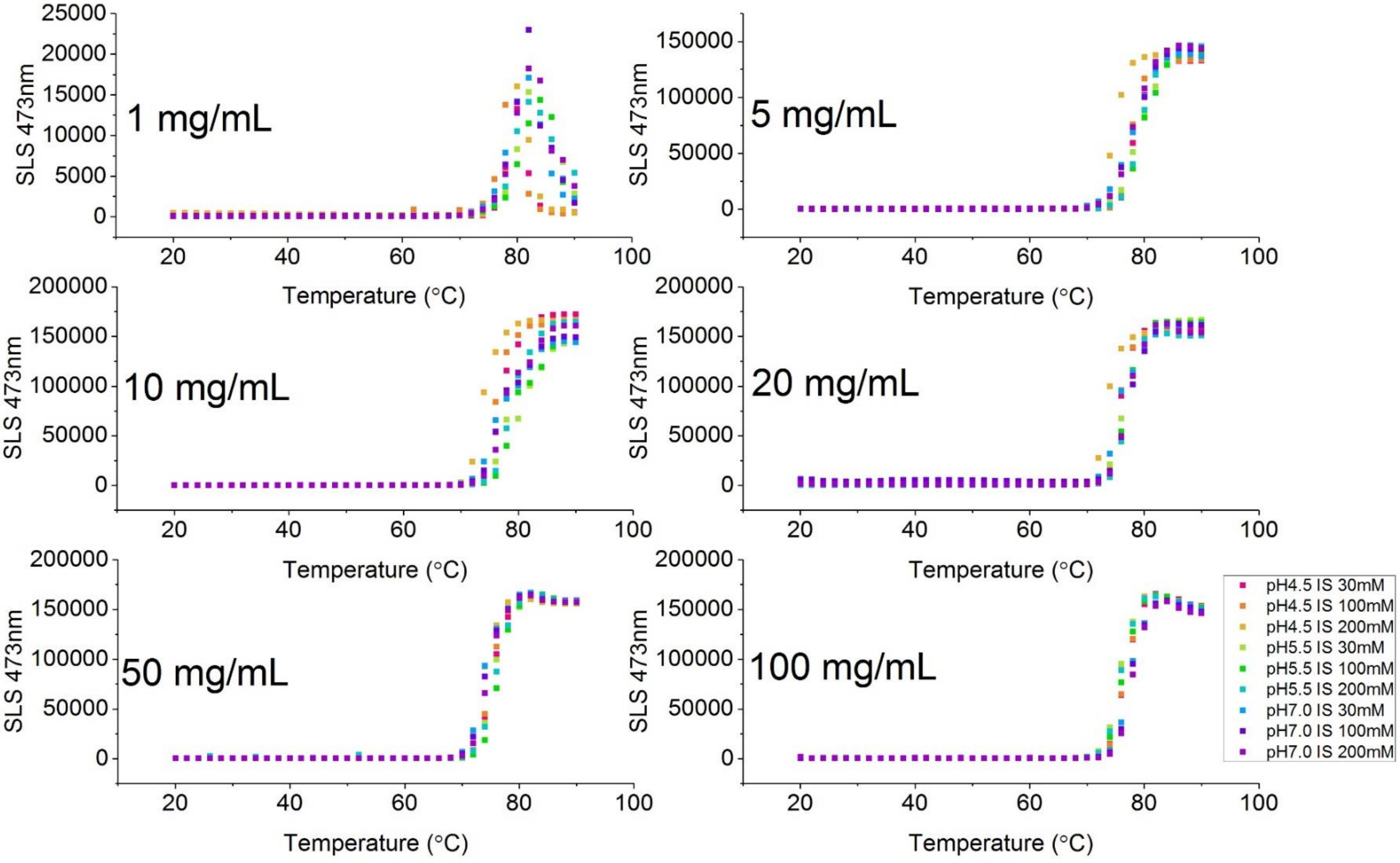


FIGURE S6: Increase in the large aggregate (reported by SLS at 473 nm) with increase in temperature for Fab from 1 to 100 mg/mL.

| pH | IS (mM) | Wavelength (nm) | $T_m$ (Experiment, °C) | | | | | |
|---|---|---|---|---|---|---|---|---|
| | | | 1 mg/mL | 5 mg/mL | 10 mg/mL | 20 mg/mL | 50 mg/mL | 100 mg/mL |
| 4.5 | 30 | 330.91 | 78.2182 | 80.3888 | 79.78 | 78.5977 | 78.008 | 78.97 |
| | | 331.86 | 78.1985 | 80.395 | 79.7795 | 78.6004 | 78.0187 | 78.9899 |
| | | 332.81 | 78.1905 | 80.42 | 79.7855 | 78.6006 | 78.0606 | 79.007 |
| | | 333.76 | 78.2013 | 80.4171 | 79.7884 | 78.6029 | 78.0929 | 79.012 |
| | | 334.71 | 78.1851 | 80.4179 | 79.7938 | 78.604 | 78.0929 | 79.0491 |
| | | 335.65 | 78.1741 | 80.4251 | 79.7945 | 78.6051 | 78.1318 | 79.0342 |
| | | 336.6 | 78.174 | 80.4345 | 79.7967 | 78.6081 | 78.1768 | 79.0834 |
| | | 337.55 | 78.1653 | 80.4655 | 79.7977 | 78.6078 | 78.2274 | 79.0978 |
| | | 338.5 | 78.172 | 80.4591 | 79.8013 | 78.6092 | 78.2227 | 79.0982 |
| | | 339.45 | 78.1601 | 80.4828 | 79.8058 | 78.61 | 78.2767 | 79.1244 |
| | | 340.39 | 78.1535 | 80.4957 | 79.8086 | 78.6109 | 78.2908 | 79.1607 |
| | | 341.34 | 78.1533 | 80.4996 | 79.8106 | 78.6125 | 78.3237 | 79.1463 |
| | | 342.29 | 78.1541 | 80.4988 | 79.8122 | 78.6126 | 78.3606 | 79.1778 |
| | | 343.23 | 78.1538 | 80.5204 | 79.8128 | 78.6138 | 78.3658 | 79.204 |
| | | 344.18 | 78.1387 | 80.529 | 79.8185 | 78.6148 | 78.4253 | 79.2069 |
| | | 345.13 | 78.1409 | 80.5278 | 79.8196 | 78.6158 | 78.4134 | 79.2437 |
| | | 346.07 | 78.1444 | 80.5352 | 79.8214 | 78.6161 | 78.4998 | 79.2508 |
| | | 347.02 | 78.1431 | 80.5509 | 79.8274 | 78.6171 | 78.4934 | 79.2688 |
| | | 347.96 | 78.1426 | 80.5518 | 79.8248 | 78.618 | 78.5433 | 79.2706 |
| | | 348.91 | 78.1432 | 80.5684 | 79.828 | 78.6181 | 78.5773 | 79.294 |
| | | 349.86 | 78.1359 | 80.5759 | 79.8316 | 78.6187 | 78.6058 | 79.2978 |
| | 100 | 330.91 | 77.7964 | 80.0357 | 77.8936 | 79.0587 | 76.4788 | 78.5425 |
| | | 331.86 | 77.8319 | 80.0354 | 77.9851 | 78.6069 | 76.4949 | 78.5467 |
| | | 332.81 | 77.7315 | 80.0353 | 77.9623 | 78.2984 | 76.5238 | 78.5726 |
| | | 333.76 | 77.7367 | 80.035 | 78.0218 | 78.4827 | 76.5568 | 78.59 |
| | | 334.71 | 77.6703 | 80.0348 | 77.9977 | 78.5238 | 76.5653 | 78.6001 |
| | | 335.65 | 77.6669 | 80.0347 | 78.0239 | 78.4635 | 76.6032 | 78.6052 |
| | | 336.6 | 77.5256 | 80.0345 | 78.0526 | 78.3529 | 76.623 | 78.6549 |
| | | 337.55 | 77.4984 | 80.0348 | 78.1101 | 78.3273 | 76.6536 | 78.6521 |
| | | 338.5 | 77.2603 | 80.0349 | 78.1484 | 78.3105 | 76.683 | 78.6684 |
| | | 339.45 | 77.0622 | 80.035 | 78.1715 | 78.318 | 76.7223 | 78.6849 |
| | | 340.39 | 76.9705 | 80.0351 | 78.1465 | 78.3574 | 76.7428 | 78.6994 |
| | | 341.34 | 76.9056 | 80.0351 | 78.2279 | 78.5653 | 76.7586 | 78.7281 |
| | | 342.29 | 76.8113 | 80.035 | 78.3016 | 78.8365 | 76.7875 | 78.7348 |
| | | 343.23 | 76.7302 | 80.0353 | 78.2812 | 78.992 | 76.8052 | 78.7563 |
| | | 344.18 | 76.6798 | 80.0355 | 78.3335 | 79.3122 | 76.832 | 78.7741 |
| | | 345.13 | 76.6779 | 80.0359 | 78.3428 | 79.2687 | 76.8595 | 78.7844 |
| | | 346.07 | 76.634 | 80.036 | 78.4516 | 79.5209 | 76.8861 | 78.8158 |
| | | 347.02 | 76.6067 | 80.0366 | 78.4509 | 79.5856 | 76.9068 | 78.8329 |
| | | 347.96 | 76.5831 | 80.0368 | 78.4971 | 79.652 | 76.9277 | 78.8484 |
| | | 348.91 | 76.567 | 80.0368 | 78.5168 | 79.6984 | 76.9537 | 78.8389 |
| | | 349.86 | 76.5427 | 80.0379 | 78.6377 | 79.7182 | 76.9905 | 78.8688 |
| | 200 | 330.91 | 78.4619 | 78.4337 | 76.2139 | 76.3485 | 75.1231 | 77.7459 |

| | | | | | | | | |
|---|---|---|---|---|---|---|---|---|
| | | 331.86 | 78.4482 | 78.433 | 76.2083 | 76.3488 | 75.1247 | 77.7595 |
| | | 332.81 | 78.4421 | 78.4281 | 76.2059 | 76.3606 | 75.18 | 77.7927 |
| | | 333.76 | 78.4317 | 78.4241 | 76.2036 | 76.3713 | 75.2029 | 77.8108 |
| | | 334.71 | 78.4194 | 78.4175 | 76.202 | 76.3733 | 75.2372 | 77.8093 |
| | | 335.65 | 78.413 | 78.4229 | 76.2005 | 76.3857 | 75.2597 | 77.8507 |
| | | 336.6 | 78.3801 | 78.4224 | 76.1988 | 76.3982 | 75.3004 | 77.8663 |
| | | 337.55 | 78.3872 | 78.4155 | 76.199 | 76.4018 | 75.3196 | 77.8813 |
| | | 338.5 | 78.3716 | 78.4162 | 76.1979 | 76.4026 | 75.3555 | 77.9036 |
| | | 339.45 | 78.3647 | 78.4119 | 76.1988 | 76.4082 | 75.3716 | 77.9131 |
| | | 340.39 | 78.3418 | 78.4124 | 76.1991 | 76.4174 | 75.4198 | 77.9296 |
| | | 341.34 | 78.3469 | 78.4067 | 76.1986 | 76.4266 | 75.4149 | 77.9462 |
| | | 342.29 | 78.3497 | 78.4094 | 76.1997 | 76.4301 | 75.4697 | 77.9739 |
| | | 343.23 | 78.3417 | 78.4069 | 76.2 | 76.4372 | 75.4955 | 77.998 |
| | | 344.18 | 78.3435 | 78.4051 | 76.2008 | 76.4394 | 75.5289 | 77.9984 |
| | | 345.13 | 78.3463 | 78.3991 | 76.2012 | 76.4441 | 75.5602 | 78.0214 |
| | | 346.07 | 78.3566 | 78.4053 | 76.2019 | 76.4513 | 75.5852 | 78.0561 |
| | | 347.02 | 78.36 | 78.4057 | 76.2033 | 76.4523 | 75.6087 | 78.0725 |
| | | 347.96 | 78.3733 | 78.4036 | 76.2037 | 76.457 | 75.6422 | 78.0809 |
| | | 348.91 | 78.4002 | 78.4012 | 76.204 | 76.4599 | 75.6983 | 78.1077 |
| | | 349.86 | 78.4047 | 78.3929 | 76.205 | 76.4658 | 75.7108 | 78.1147 |
| 5.5 | 30 | 330.91 | 81.0 | 87.0 | 86.3262 | 80.7735 | 81.0743 | 78.1271 |
| | | 331.86 | 81.0 | 87.0 | 86.7722 | 80.8474 | 81.1322 | 78.1685 |
| | | 332.81 | 81.0 | 87.0 | 86.252 | 80.8913 | 81.145 | 78.1661 |
| | | 333.76 | 81.0 | 87.0 | 86.2224 | 80.9147 | 81.1406 | 78.2008 |
| | | 334.71 | 81.0 | 87.0 | 86.2291 | 80.9389 | 81.2326 | 78.2268 |
| | | 335.65 | 81.0 | 87.0 | 86.2112 | 80.954 | 81.2211 | 78.2395 |
| | | 336.6 | 81.0 | 87.0 | 86.1744 | 80.9835 | 81.2729 | 78.2957 |
| | | 337.55 | 81.0 | 87.0 | 86.2424 | 81.0216 | 81.3454 | 78.2975 |
| | | 338.5 | 81.0 | 87.0 | 86.1867 | 81.0352 | 81.3616 | 78.3026 |
| | | 339.45 | 81.0 | 87.0 | 86.1397 | 81.0834 | 81.3233 | 78.3207 |
| | | 340.39 | 81.0 | 86.9674 | 86.1291 | 81.1471 | 81.3987 | 78.354 |
| | | 341.34 | 81.0 | 87.0 | 86.1252 | 81.1646 | 81.4051 | 78.3716 |
| | | 342.29 | 81.0 | 86.9102 | 86.0896 | 81.1931 | 81.4863 | 78.3848 |
| | | 343.23 | 81.0 | 86.9051 | 86.0893 | 81.2539 | 81.4865 | 78.3966 |
| | | 344.18 | 81.0 | 86.8925 | 86.0764 | 81.3043 | 81.5777 | 78.4239 |
| | | 345.13 | 81.0 | 86.8835 | 86.065 | 81.331 | 81.5746 | 78.4495 |
| | | 346.07 | 81.0 | 86.7954 | 86.0621 | 81.3445 | 81.6248 | 78.4427 |
| | | 347.02 | 81.0 | 86.7974 | 86.0461 | 81.3688 | 81.6948 | 78.458 |
| | | 347.96 | 81.0 | 86.7526 | 86.0439 | 81.4044 | 81.7185 | 78.496 |
| | | 348.91 | 81.0 | 86.7394 | 86.0379 | 81.4556 | 81.7383 | 78.5109 |
| | | 349.86 | 81.0 | 86.7079 | 86.0322 | 81.4686 | 81.7259 | 78.5533 |
| | 100 | 330.91 | 82.0 | 86.0005 | 87.0 | 79.7325 | 79.4025 | 78.3937 |
| | | 331.86 | 82.0 | 86.0131 | 87.0 | 79.7544 | 79.4566 | 78.4368 |
| | | 332.81 | 82.0 | 85.9993 | 87.0 | 79.7585 | 79.4771 | 78.4451 |
| | | 333.76 | 82.0 | 85.9965 | 87.0 | 79.783 | 79.5413 | 78.4767 |

| | | | | | | | | | |
|---|---|---|---|---|---|---|---|---|---|
| | | 334.71 | 82.0 | 85.9917 | 87.0 | 79.794 | 79.5369 | 78.4809 |
| | | 335.65 | 82.0 | 85.9604 | 87.0 | 79.8125 | 79.6176 | 78.4966 |
| | | 336.6 | 82.0 | 85.9425 | 87.0 | 79.8203 | 79.6304 | 78.5212 |
| | | 337.55 | 82.0 | 85.9472 | 87.0 | 79.8594 | 79.6685 | 78.5326 |
| | | 338.5 | 82.0 | 85.9133 | 87.0 | 79.8529 | 79.6833 | 78.55 |
| | | 339.45 | 82.0 | 85.8936 | 87.0 | 79.8678 | 79.7604 | 78.5745 |
| | | 340.39 | 82.0 | 85.8946 | 87.0 | 79.885 | 79.7458 | 78.5812 |
| | | 341.34 | 82.0 | 85.8659 | 87.0 | 79.9073 | 79.7352 | 78.6023 |
| | | 342.29 | 82.0 | 85.8466 | 87.0 | 79.9197 | 79.8398 | 78.6029 |
| | | 343.23 | 82.0 | 85.8553 | 87.0 | 79.9471 | 79.8338 | 78.6722 |
| | | 344.18 | 82.0 | 85.8357 | 87.0 | 79.9437 | 79.8895 | 78.6586 |
| | | 345.13 | 82.0 | 85.8213 | 87.0 | 79.978 | 79.9542 | 78.6773 |
| | | 346.07 | 82.0 | 85.8029 | 87.0 | 79.9832 | 79.9776 | 78.7095 |
| | | 347.02 | 82.0 | 85.8031 | 86.9999 | 79.9968 | 79.9674 | 78.7196 |
| | | 347.96 | 82.0 | 85.8004 | 86.9997 | 80.0108 | 80.0178 | 78.7331 |
| | | 348.91 | 82.0 | 85.7818 | 86.9996 | 80.0311 | 80.0478 | 78.7469 |
| | | 349.86 | 82.0 | 85.7729 | 86.9999 | 80.0451 | 80.09 | 78.7773 |
| | 200 | 330.91 | 80.0 | 84.8912 | 79.9219 | 78.9006 | 78.7988 | 77.7823 |
| | | 331.86 | 80.0 | 84.8473 | 79.9428 | 78.9211 | 78.8324 | 77.7975 |
| | | 332.81 | 80.0 | 84.8373 | 79.9622 | 78.9379 | 78.8297 | 77.8215 |
| | | 333.76 | 80.0 | 84.8298 | 79.9821 | 78.9835 | 78.8584 | 77.8389 |
| | | 334.71 | 80.0 | 84.8251 | 79.9984 | 79.0326 | 78.8585 | 77.8609 |
| | | 335.65 | 80.0 | 84.8013 | 80.0095 | 79.0343 | 78.9222 | 77.8715 |
| | | 336.6 | 80.0 | 84.7935 | 80.0264 | 79.0919 | 78.9403 | 77.8953 |
| | | 337.55 | 80.0 | 84.8229 | 80.0405 | 79.0989 | 78.9861 | 77.9213 |
| | | 338.5 | 80.0 | 84.7692 | 80.053 | 79.1331 | 78.9522 | 77.9423 |
| | | 339.45 | 80.0 | 84.7733 | 80.0724 | 79.1417 | 79.032 | 77.94 |
| | | 340.39 | 80.0 | 84.7562 | 80.0815 | 79.1549 | 79.0635 | 77.9745 |
| | | 341.34 | 80.0 | 84.7761 | 80.0963 | 79.203 | 79.1127 | 77.9731 |
| | | 342.29 | 80.0 | 84.7686 | 80.1076 | 79.2246 | 79.1592 | 78.0235 |
| | | 343.23 | 80.0 | 84.7716 | 80.118 | 79.2529 | 79.115 | 78.0352 |
| | | 344.18 | 80.0 | 84.7365 | 80.129 | 79.2777 | 79.1324 | 78.0329 |
| | | 345.13 | 80.0 | 84.7256 | 80.14 | 79.2986 | 79.1953 | 78.0704 |
| | | 346.07 | 80.0 | 84.7472 | 80.1528 | 79.3322 | 79.1987 | 78.0714 |
| | | 347.02 | 80.0 | 84.7334 | 80.1617 | 79.3192 | 79.2756 | 78.1036 |
| | | 347.96 | 80.0 | 84.7552 | 80.1659 | 79.3641 | 79.3331 | 78.1459 |
| | | 348.91 | 80.0 | 84.7458 | 80.178 | 79.3717 | 79.3288 | 78.1412 |
| | | 349.86 | 80.0 | 84.7509 | 80.1846 | 79.4093 | 79.3626 | 78.1632 |
| 7.0 | 30 | 330.91 | 79.087 | 84.5064 | 84.216 | 79.6113 | 78.3364 | 80.1066 |
| | | 331.86 | 79.148 | 84.4461 | 84.216 | 79.6455 | 78.3631 | 80.1421 |
| | | 332.81 | 79.1591 | 84.3649 | 84.216 | 79.6641 | 78.426 | 80.1597 |
| | | 333.76 | 79.1567 | 84.2937 | 84.216 | 79.6802 | 78.4294 | 80.1723 |
| | | 334.71 | 79.1275 | 84.2133 | 84.216 | 79.7006 | 78.4619 | 80.1996 |
| | | 335.65 | 79.1879 | 84.1584 | 84.216 | 79.7174 | 78.4882 | 80.2165 |
| | | 336.6 | 79.1965 | 84.0736 | 84.216 | 79.7474 | 78.5394 | 80.255 |

| | | | | | | | | |
|---|---|---|---|---|---|---|---|---|
| | | 337.55 | 79.185 | 84.0651 | 84.216 | 79.7831 | 78.572 | 80.2535 |
| | | 338.5 | 79.2491 | 84.0134 | 84.216 | 79.7985 | 78.5698 | 80.2666 |
| | | 339.45 | 79.2001 | 83.9796 | 84.216 | 79.83 | 78.66 | 80.3014 |
| | | 340.39 | 79.2399 | 83.9191 | 84.216 | 79.8782 | 78.6418 | 80.3034 |
| | | 341.34 | 79.2416 | 83.9247 | 84.216 | 79.9079 | 78.6899 | 80.3576 |
| | | 342.29 | 79.2573 | 83.9042 | 84.216 | 79.9114 | 78.7567 | 80.3601 |
| | | 343.23 | 79.3128 | 83.8663 | 84.216 | 79.9669 | 78.7961 | 80.395 |
| | | 344.18 | 79.2823 | 83.8114 | 84.216 | 79.978 | 78.7941 | 80.3781 |
| | | 345.13 | 79.2787 | 83.832 | 84.216 | 79.9977 | 78.8793 | 80.4032 |
| | | 346.07 | 79.2816 | 83.7821 | 84.216 | 80.0472 | 78.9037 | 80.4249 |
| | | 347.02 | 79.3203 | 83.7652 | 84.216 | 80.0865 | 78.9197 | 80.445 |
| | | 347.96 | 79.3249 | 83.7407 | 84.216 | 80.0891 | 78.9676 | 80.4839 |
| | | 348.91 | 79.3569 | 83.7303 | 84.216 | 80.1184 | 78.9822 | 80.4901 |
| | | 349.86 | 79.3711 | 83.7185 | 84.216 | 80.1533 | 78.9881 | 80.5227 |
| | 100 | 330.91 | 79.9961 | 83.7284 | 86.8187 | 80.9124 | 77.8258 | 80.249 |
| | | 331.86 | 79.9912 | 83.6915 | 86.8331 | 80.9416 | 77.8646 | 80.2639 |
| | | 332.81 | 79.9329 | 83.6717 | 86.7105 | 80.9542 | 77.8874 | 80.3162 |
| | | 333.76 | 79.9008 | 83.6582 | 86.6253 | 80.9681 | 77.9062 | 80.328 |
| | | 334.71 | 79.9075 | 83.6461 | 86.4592 | 80.99 | 77.9206 | 80.3575 |
| | | 335.65 | 79.9161 | 83.6266 | 86.5342 | 80.9762 | 77.9886 | 80.3579 |
| | | 336.6 | 79.9651 | 83.6089 | 86.3473 | 81.0257 | 77.9953 | 80.368 |
| | | 337.55 | 79.9187 | 83.573 | 86.2935 | 81.0197 | 78.0046 | 80.3997 |
| | | 338.5 | 79.9378 | 83.5721 | 86.2948 | 81.0567 | 78.054 | 80.4259 |
| | | 339.45 | 79.9788 | 83.5469 | 86.2342 | 81.0687 | 78.1082 | 80.432 |
| | | 340.39 | 79.983 | 83.5305 | 86.2057 | 81.0874 | 78.1087 | 80.4522 |
| | | 341.34 | 80.0 | 83.525 | 86.1727 | 81.1127 | 78.1677 | 80.4694 |
| | | 342.29 | 80.0 | 83.4981 | 86.0441 | 81.1225 | 78.1812 | 80.4817 |
| | | 343.23 | 80.0 | 83.4879 | 86.0631 | 81.1316 | 78.2061 | 80.5036 |
| | | 344.18 | 80.0 | 83.469 | 85.9864 | 81.1748 | 78.2554 | 80.5266 |
| | | 345.13 | 80.0 | 83.455 | 85.9608 | 81.1782 | 78.2986 | 80.5565 |
| | | 346.07 | 80.0 | 83.4445 | 85.9458 | 81.1848 | 78.3504 | 80.5594 |
| | | 347.02 | 80.0 | 83.437 | 85.8933 | 81.2094 | 78.3802 | 80.6081 |
| | | 347.96 | 80.0 | 83.4472 | 85.8451 | 81.205 | 78.3869 | 80.6277 |
| | | 348.91 | 80.0 | 83.4086 | 85.8448 | 81.2241 | 78.435 | 80.6306 |
| | | 349.86 | 80.0 | 83.3998 | 85.789 | 81.2605 | 78.4639 | 80.6557 |
| | 200 | 330.91 | 81.0 | 83.3385 | 86.0 | 79.6785 | 76.2872 | 80.3091 |
| | | 331.86 | 81.0 | 83.325 | 86.0 | 79.6893 | 76.3014 | 80.2929 |
| | | 332.81 | 81.0 | 83.3039 | 86.0 | 79.6898 | 76.3359 | 80.3666 |
| | | 333.76 | 81.0 | 83.2794 | 86.0 | 79.7154 | 76.3693 | 80.3708 |
| | | 334.71 | 81.0 | 83.273 | 86.0 | 79.7232 | 76.4099 | 80.3819 |
| | | 335.65 | 81.0 | 83.2772 | 86.0 | 79.7397 | 76.4213 | 80.4324 |
| | | 336.6 | 81.0 | 83.2802 | 86.0 | 79.752 | 76.4539 | 80.4237 |
| | | 337.55 | 81.0 | 83.2566 | 86.0 | 79.779 | 76.4578 | 80.4356 |
| | | 338.5 | 81.0 | 83.2722 | 86.0 | 79.7665 | 76.5092 | 80.4751 |
| | | 339.45 | 81.0 | 83.2552 | 85.9541 | 79.8005 | 76.5611 | 80.4825 |

| | | | | | | | | |
|---|---|---|---|---|---|---|---|---|
| | | 340.39 | 81.0 | 83.2368 | 85.9424 | 79.8199 | 76.5745 | 80.4844 |
| | | 341.34 | 81.0 | 83.2448 | 85.9213 | 79.8237 | 76.587 | 80.5175 |
| | | 342.29 | 81.0 | 83.2403 | 85.9096 | 79.8285 | 76.612 | 80.5368 |
| | | 343.23 | 81.0 | 83.2141 | 85.8961 | 79.8454 | 76.6606 | 80.5379 |
| | | 344.18 | 81.0 | 83.2242 | 85.8571 | 79.8754 | 76.6862 | 80.543 |
| | | 345.13 | 81.0 | 83.2367 | 85.8705 | 79.883 | 76.7365 | 80.6118 |
| | | 346.07 | 81.0 | 83.1968 | 85.8606 | 79.8937 | 76.7422 | 80.6024 |
| | | 347.02 | 81.0 | 83.2047 | 85.8791 | 79.9116 | 76.7707 | 80.6572 |
| | | 347.96 | 81.0 | 83.1939 | 85.8608 | 79.9154 | 76.821 | 80.6554 |
| | | 348.91 | 81.0 | 83.2014 | 85.8863 | 79.9448 | 76.8546 | 80.6798 |
| | | 349.86 | 81.0 | 83.1941 | 85.8938 | 79.9503 | 76.8892 | 80.7182 |

Table S3: Unfolding midpoint ($T_m$) of Fab corresponding to each wavelength between 330-350 nm, obtained from the global fitting of the denaturation curves allowing the midpoint to vary.

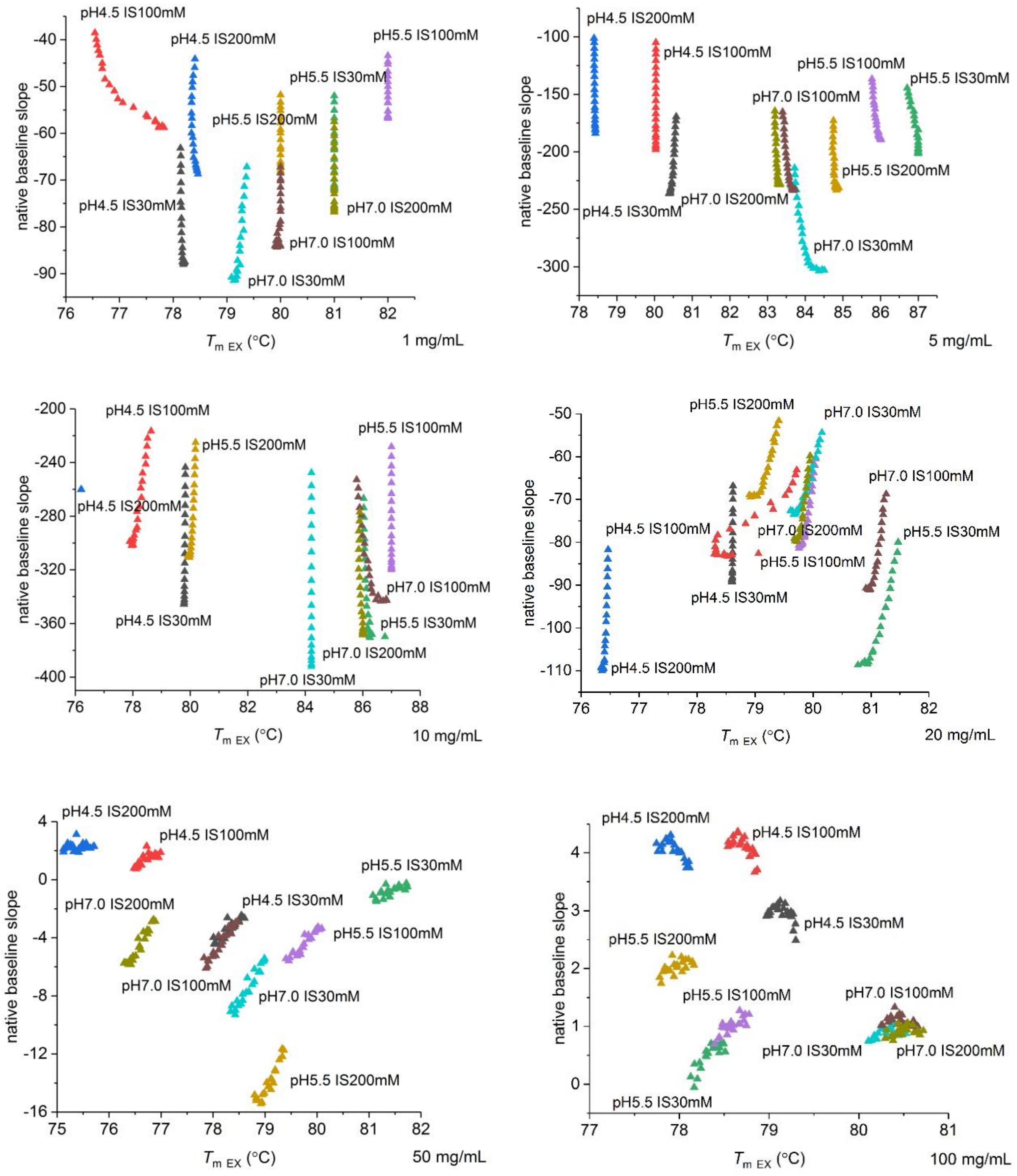


FIGURE S7: Slope of the native baseline of Fab fluorescence from global fitting of the denaturation curves. (pH4.5 IS 30mM: ▲, pH4.5 IS 100mM: ▲, pH4.5 IS 200mM: ▲, pH5.5 IS 30mM: ▲, pH5.5 IS 100mM: ▲, pH5.5 IS 200mM: ▲, pH7.0 IS 30mM: ▲, pH7.0 IS 100mM: ▲, pH7.0 IS 200mM: ▲). Each buffer condition shows the 21 datapoints for the baseline slopes of the denaturation curves obtained from spectra at every 1 nm in the 330-350 nm range.

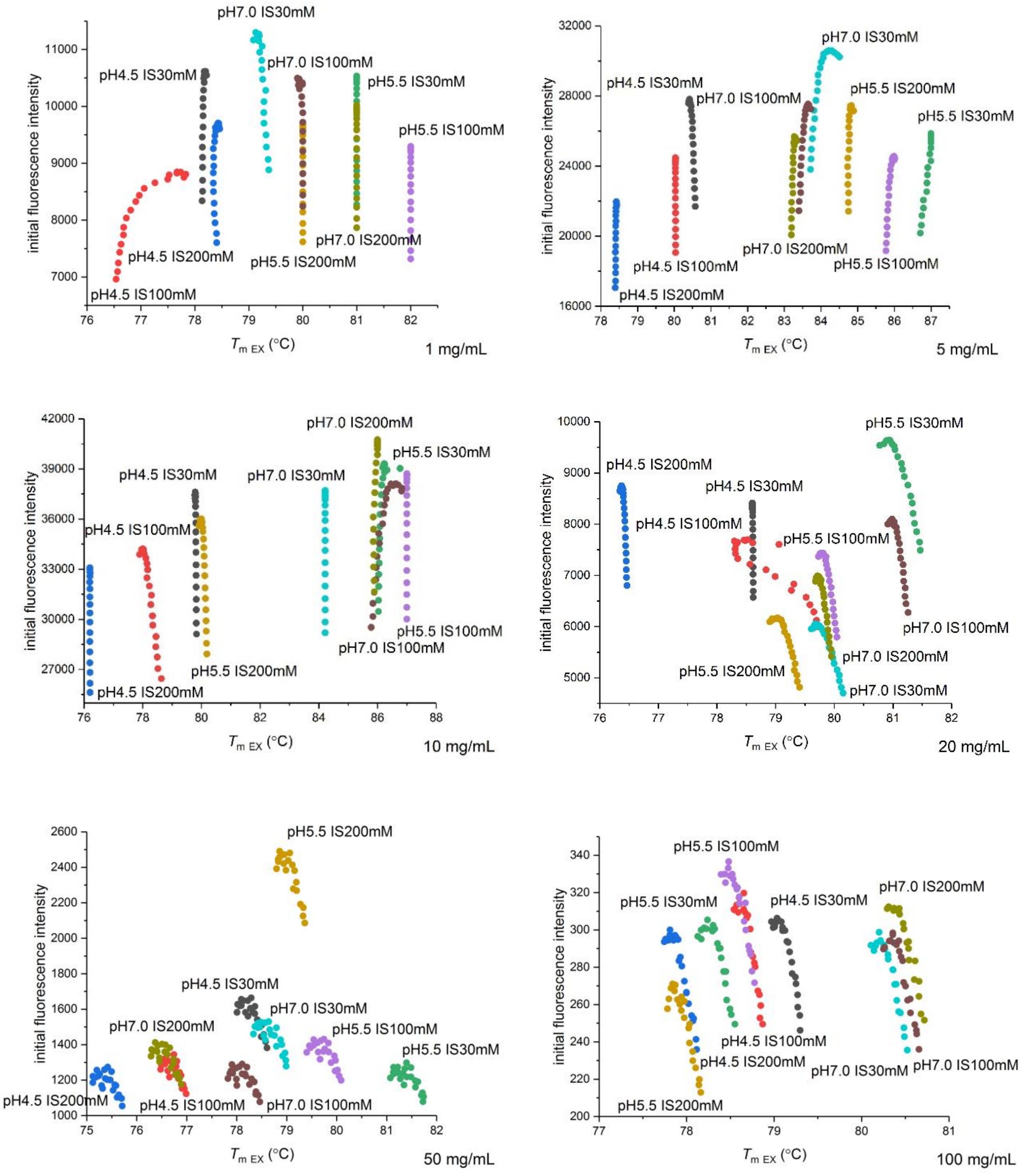


FIGURE S8: Initial fluorescence intensity of Fab from global fitting of the denaturation curves (pH4.5 IS 30mM: ●, pH4.5 IS 100mM: ●, pH4.5 IS 200mM: ●, pH5.5 IS 30mM: ●, pH5.5 IS 100mM: ●, pH5.5 IS 200mM: ●, pH7.0 IS 30mM: ●, pH7.0 IS 100mM: ●, pH7.0 IS 200mM: ●). Each buffer condition shows the 21 datapoints for the initial fluorescence (i.e. at 20 °C), for the denaturation curves obtained from spectra at every 1 nm in the 330-350 nm range.
